# Evaluating self- and informant-reported complaints as predictors of cognitive progression in subjective cognitive decline: A clinically-based evaluation

**DOI:** 10.1177/13872877261443656

**Published:** 2026-04-23

**Authors:** Hana Horakova, Dylan J. Jester, Terezie Vohradkova, Katerina Sheardova, Jan Ondrej, Veronika Matuskova, Adela Fendrych Mazancova, Tomas Nikolai, Ross Andel, Ondrej Lerch, Jan Laczó, Jakub Hort, Martin Vyhnalek

**Affiliations:** 1 Memory Clinic, Department of Neurology, Second Faculty of Medicine, 37740 Charles University and Motol University Hospital, Prague, Czech Republic; 2 Department of Clinical Psychology, 48359 Motol University Hospital, Prague, Czech Republic; 3 Department of Psychology, Faculty of Arts, 112300 Charles University, Prague, Czech Republic; 4 War Related Illness and Injury Study Center (WRIISC), 19977 VA Palo Alto Health Care System, Palo Alto, CA, USA; 5 Edson College of Nursing and Health Innovation, Arizona State University, Phoenix, AZ, USA; 6 International Clinical Research Center, St Anne's University Hospital Brno, Brno, Czech Republic

**Keywords:** Alzheimer's disease, cognition, cognitive aging, cognitive dysfunction, dementia, longitudinal studies, mild cognitive impairment, pathological aging

## Abstract

**Background:**

Subjective cognitive complaints (SCCs) in cognitively normal older adults are associated with increased dementia risk. However, it remains uncertain which complaints best predict cognitive decline and whether predictive value differs between patient and informant reports.

**Objective:**

To examine associations between self- and informant-reported total scores and individual items from the Questionnaire of Cognitive Complaints (QPC) and longitudinal cognitive decline in individuals with subjective cognitive decline (SCD).

**Methods:**

261 individuals with SCD from the prospective Czech Brain Aging Study were followed longitudinally (mean 4.2-year follow-up). Linear mixed-effects models (random intercepts), adjusted for age, sex, and education, were estimated separately for self- and informant-reported QPC-total scores and for individual QPC-items to examine associations with change over time in global cognition, memory, and executive function/processing speed cognitive composites.

**Results:**

Higher QPC-total scores predicted steeper decline across all cognitive composites in both self- and informant-reports, with modest effects. At the item level, after Bonferroni correction, informant-reported *impressions of memory change* and *worse memory compared with peers* were associated with a more pronounced decline across all cognitive composites exceeding the predictive value of the QPC-total score. Several other memory- and non-memory-related complaints showed associations in the same direction but did not consistently remain significant after correction.

**Conclusions:**

In older adults with SCD, focusing on specific SCCs, particularly informant-reported memory complaints, rather than relying solely on global SCC score, allows more targeted identification of individuals whose complaints are most consistently associated with longitudinal cognitive decline. Structured assessments integrating informant perspective may improve early risk stratification.

## Introduction

Subjective cognitive complaints (SCCs) are commonly reported by older adults and may reflect normal aging processes, but they may also be associated with cognitive impairment.^1^ Even in cognitively normal individuals, SCCs have been consistently linked to a higher risk of subsequent cognitive decline and the development of dementia,^2^ particularly among those who seek medical consultation for such concerns.^3^

Among several neurodegenerative and non-neurodegenerative conditions associated with dementia, Alzheimer's disease (AD) is the most prevalent, with pathological brain changes occurring long before noticeable symptoms appear. During early stages of these conditions, including the preclinical stage of AD, some individuals may perceive subtle cognitive changes and spontaneously report them as SCCs, despite performing within the normal cognitive range.

To enhance the understanding and investigation of SCCs in cognitively normal older adults, the concept of subjective cognitive decline (SCD) has been introduced.^4^ SCD refers to a self-perceived, persistent decline in cognitive abilities over the past five years compared to an individual's previous functioning, which is not attributable to an acute medical event or treatment effects, and occurs despite cognitive test performance remaining comparable to that of demographically matched healthy individuals.^1,4^

While in some studies SCCs have been linked to neuroimaging and metabolic biomarkers associated with AD,^5–9^ they are not AD-specific and may also reflect other neurodegenerative and non-neurodegenerative processes, including vascular changes.^1^ Given these complexities, increasing attention is being devoted to analyzing specific SCCs. Notably, previous studies have identified memory-related complaints, such as *progressive change in memory* or *worse memory in comparison to peers*, as particularly linked to a higher risk of pathological cognitive decline beyond normal aging.^8,10–13^ However, these findings may partly reflect the research focus, as most studies have primarily examined memory-related complaints,^1,14^ despite evidence that early pathological processes may also manifest through changes in other cognitive domains,^15,16^ as well as through subtle behavioral or functional concerns.^17^

Another important aspect of SCCs, in terms of their diagnostic and predictive validity, is determining whose reports should be considered. Beyond self-reports, informant reports are increasingly recognized as valuable for assessing patients’ cognitive status and their risk of clinical progression.^18^ Yet, the relative significance and interpretation of each of these sources of reports in cognitively normal older adults remain unclear.

A recent meta-analysis^19^ found that both self- and informant-reported SCCs predicted clinical transition. However, in terms of the strength of the association, informant-reported complaints were generally more strongly linked to the progression toward cognitive impairment. Additional evidence suggests that the magnitude of this predictive advantage is not uniform and appears to increase with advancing cognitive decline.^20,21^ In this context, Caselli et al.^17^ found that self-endorsed decline preceded informant-reported decline in cognitively normal older adults who later developed mild cognitive impairment (MCI). This finding suggests that the relevance of self- and informant-reported SCCs may dynamically change along the cognitive trajectory, with self-reports potentially capturing the earliest subtle changes that may remain undetected by informants for some time.

Another notable issue requiring further clarification regarding specific SCCs is whether the distinct profiles reported by participants and informants offer differential insights into cognitive changes over time, an issue that has emerged from findings of a few prior studies.^18^ Studies directly comparing the relevance of specific SCCs based on their source are scarce and predominantly community-based.^22,23^ In particular, evidence regarding the role of self- and informant-reported SCCs in predicting cognitive outcomes in clinically ascertained populations with baseline SCD remains limited.^3,21^

Currently, no standardized approach exists for assessing SCCs. In our previous cross-sectional studies,^11,12^ we used the Questionnaire of Cognitive Complaints (QPC; from French *Le Questionnaire de Plainte Cognitive*), a brief and easy-to-administer tool encompassing several distinct complaints previously associated with increased risk of pathological aging^24,25^: 1) memory decline relative to previous ability; 2) memory functioning compared to age-matched individuals; 3) other specific complaints encompassing both memory- and non-memory-related difficulties; and 4) two additional items reflecting functional and behavioral concerns, namely limitations in daily activities and perceived personality change. Our findings indicated that specific QPC complaints, rather than total score, were more informative in identifying individuals at risk. However, their clinical utility remains limited without longitudinal validation.

Building on our previous research^11,12^ and addressing ongoing questions regarding the validity of self- and informant-reported SCCs in predicting clinical progression,^18^ this longitudinal study aimed to assess the associations between self- and informant-reported SCCs in the QPC and cognitive decline over time. Specifically, we analyzed both the self- and informant-reported QPC-total score and specific QPC-items, to determine their ability to predict trajectories of decline in global cognition, memory and executive function/processing speed in a clinically ascertained SCD sample over an average follow-up of 4.2 years. Based on prior research, we hypothesized that (a) specific SCCs would be associated with greater cognitive decline, and (b) informant reports would show relatively stronger associations compared to self-reports. Additionally, we expected (c) that specific SCCs may show differential associations with cognitive decline depending on the source of the report (self versus informant).

## Methods

### Participants

The study sample consisted of 261 older adults with SCD who participated in the Memory Clinic-based Czech Brain Aging Study (CBAS).^26^ The participants were recruited and followed prospectively with at least one follow-up examination at one of the CBAS centers: (a) Memory Clinic, Department of Neurology, Second Faculty of Medicine, Charles University and Motol University Hospital, Prague (n = 67); (b) Memory Center by International Clinical Research Center, St Anne's University Hospital, Brno (n = 194). All individuals were referred to the memory clinics by general practitioners, neurologists, psychiatrists, or geriatricians based on SCCs reported by themselves and/or their informants. All participants underwent a standard diagnostic workup, including neurological and laboratory evaluations, brain magnetic resonance imaging (MRI; 1.5 or 3 T with MPRAGE sequences) and comprehensive neuropsychological examination at baseline, and were followed prospectively with clinical and neuropsychological evaluations in order to monitor cognitive performance over time.

After the initial visit, participants were examined one year later, then regularly on an approximately biannual basis. Additional visits were available if significant changes were reported by the patient or by the informant. Follow-up ended upon progression to dementia. During the period of observation, 7 (4.3%) individuals converted to dementia. The mean and median time to conversion was 3.2 and 3.4 years, respectively. The diagnosis of dementia was based on clinical history reported by the patient and the informant, neurological examination, neuropsychological assessment, and MRI. Dementia was diagnosed when objective cognitive impairment was accompanied by impairment of activities of daily living resulting in loss of independence, as reported by the patient's informant. This functional impairment distinguished dementia from pre-dementia stages.^27^

General exclusion criteria for CBAS include a history or evidence of neurological conditions potentially affecting cognition, such as stroke, traumatic brain injury, or neurodegenerative diseases other than AD; psychiatric disorders, including major depressive disorder, bipolar affective disorder, generalized anxiety disorder, schizophrenia, or substance abuse; and systemic or other medical conditions that could potentially impact cognition.^26^

All participants provided written informed consent approved by local ethics committees (Motol University Hospital; St Anne's University Hospital Brno), and all procedures followed the Helsinki Declaration.

### Definition of SCD

All individuals met published criteria for SCD at baseline,^4^ including a self-experienced persistent decline in cognitive capacity within the last 5 years when compared to their previously normal status and unrelated to an acute event, and normal performance on standardized cognitive tests. The nature and intensity of the self-experienced cognitive decline was ascertained by a semi-structured interview with an experienced clinician. Normal performance was defined as no single score more than 1.5 standard deviations (SD) below the mean of age- and education-adjusted norms, based on comprehensive neuropsychological battery (see *Measures* for details). Only patients whose motivation for seeking medical consultation was self-perceived cognitive decline were included.

### Measures

#### Evaluation of SCCs: QPC

The Czech version of the QPC, a 10-item yes/no questionnaire originally developed in French to assess cognitive difficulties over the past six months,^28^ was used to measure SCCs, utilizing both the self- and informant-rated versions. The translation to Czech was described previously.^11,12^ The first two items of the QPC assess general perceptions of memory abilities, while the following six items address specific SCCs encompassing both memory- and non-memory-related difficulties, such as spatial orientation, and language. In addition, the questionnaire includes two items reflecting subjective functional and behavioral concerns, namely instrumental activities, and perceived personality changes. For each item, we indicate a key name for ease in reporting. The exact wording of all items is available elsewhere.^11,12^

#### Neuropsychological and affective assessment

The comprehensive neuropsychological test battery was based on the Czech version of the Uniform Data Set 2.0^29^ enriched by additional tests of memory, visuoconstruction and phonemic verbal fluency. Specifically, the battery included measures of: (a) global cognitive function (Mini-Mental State Examination), (b) processing speed (Digit Symbol Coding, DS-C; Trail Making Test A, TMT-A), (c) attention and working memory (Digit Span Forward, DS-F, and Backward, DS-B); (d) verbal memory (Logical memory, LM; Rey Auditory Verbal Learning Test, RAVLT); (e) non-verbal memory (Brief Visuospatial Memory Test—Revised; Rey-Osterrieth Complex Figure reproduction after 3 min, ROCFT-R); (f) language (Boston Naming Test, 30 odd-items version; semantic verbal fluency animals and vegetables, S-VF); (g) executive function (TMT-B; phonemic verbal fluency); (h) visuospatial function (ROCFT copy condition, ROCFT-C; Clock Drawing Test).

The Geriatric Depression Scale, 15-item version, and the Beck Anxiety Inventory evaluated the levels of depressive and anxiety symptomatology.

#### Measures of cognitive decline

To enhance the measurement of subtle cognitive changes, several empirically derived composite scores have been developed by combining neuropsychological tests that are sensitive to decline in the preclinical or prodromal stages of AD, with empirical data from observational studies.^30^ In this study, we used domain-specific composites for memory and executive function/processing speed, as well as a global cognitive composite, as these have previously been shown to be the most sensitive in capturing subtle but clinically meaningful cognitive changes.^30^ The selection of individual tests for each composite was guided by previous empirical findings on their sensitivity to early cognitive decline. The labels of the composite scores were selected to best approximate the underlying cognitive domains.

All composite scores were calculated following a standardized multi-step procedure. First, raw scores for each neuropsychological test were transformed into z-scores using the means and standard deviations (SDs) derived from baseline performance, which served as the reference for all composite scores of the follow-up visits. For TMT-B in which higher scores indicated poorer performance, z-scores were reversed prior to further processing. Second, domain-specific composite scores were calculated by averaging the z-scores of the corresponding tests. The *memory* composite was computed as the mean of the z-scores for LM delayed recall, RAVLT sum of trials 1 to 5, and RAVLT delayed recall. The *executive function/processing speed* composite was calculated as the mean of DS-C, TMT-B and S-VF, with S-VF represented as one single z-score evenly weighted between the animal and vegetable fluency [(z-score for animals + z-score for vegetables)/2]. Finally, the *global cognitive composite* was calculated by averaging the Mini-Mental State Examination z-score, the memory composite, and the executive function/processing speed composite. All composite scores were standardized (mean = 0, SD = 1).

### Statistical analyses

Initially, the assumption of normality was verified based on skewness and kurtosis values falling within the range of −1 to +1, allowing for the comparison of QPC-total scores between the self-rated and informant-rated versions using a paired-samples t-test, with Cohen's d calculated to quantify effect sizes. Differences in QPC-items frequencies reported by patients and their informants were assessed using continuity-corrected McNemar's test, with odds ratios (OR) as effect size.

Linear mixed effects models with a random intercept were used to examine rate of change in global cognition, memory, and executive function/processing speed composites. Each model contained baseline age, sex, and education level as covariates and time (the slope).

To understand whether greater cognitive complaints were associated with worse cognitive decline in global cognition, memory, and executive function/processing speed over time, the QPC-total score and an interaction of the QPC-total score with time were included in the linear mixed effects models. The beta coefficient for time can be interpreted as the average rate of change in the cognitive domain per follow-up assessment. The beta coefficient for QPC-total score can be interpreted as the average increase or decrease in the cognitive domain at baseline for each unit increase in the QPC-total score. Finally, the beta coefficient for the QPC-total score by time interaction can be interpreted as the additional average rate of decline for each unit increase in the QPC-total score.

To determine whether specific QPC items were associated with differential rates of decline in global cognition, memory, and executive function/processing speed, separate item-level models were estimated. In these item-specific models, only the QPC-item and its interaction with time were entered, while the QPC-total score and its interaction with time were intentionally omitted to avoid redundancy and collinearity. The beta coefficient for QPC-item can be interpreted as the average increase or decrease in the cognitive domain at baseline for patients who endorsed the item or had their informant endorse the item, and the beta coefficient for the QPC-item by time interaction can be interpreted as the additional average rate of decline for patients who endorsed the item or had their informant endorse the item.

Due to the large number of models conducted for the QPC-item analyses, p-values < 0.005 are considered statistically significant after controlling for Familywise Type I Error using a conservative Bonferroni correction.

## Results

### Characteristics of the sample

Baseline demographic, affective, and cognitive characteristics of the SCD sample (n = 261) are presented in Table 1. The mean follow-up duration for the entire sample was 4.2 years (range: 0.9–8.7 years), with a median follow-up of 4.2 years. Participants attended an average of 3.8 visits (range: 2–7) during this period, contributing an average of 11.5 measurements (range: 6–21) per individual across the follow-up timeframe.

**Table 1.** Demographic, affective and cognitive characteristics of the SCD sample.

	Mean (SD)	Median	IQR
*Demographic characteristics*			
Age	67.2 (7.4)	66	11
Education	15.0 (2.9)	14.5	5
Female, % (n)	66.3 (173)	-	-
Mean follow-up duration	4.2 (2.2)	4.2	4
Visits per participant	3.8 (1.3)	4	2
*Depressive and anxious symptoms*			
GDS-15	3.7 (2.8)	3	3
BAI	11.9 (9.2)	10	10.8
*Cognitive assessment*			
MMSE	28.9 (1.1)	29	2
DS-C	44.1 (9.0)	44	13.3
TMT A	39.2 (12.3)	37.5	13.8
TMT B	88.7 (33.4)	81.3	38
P-VF	47.0 (11.3)	46	14
S-VF-A	24.8 (5.9)	25	9
S-VF-V	13.6 (3.5)	13	5
BNT	2.4 (2.1)	2	3
CDT	15.4 (1.6)	16	1
ROCF-C	30.2 (3.6)	32	5
ROCF-R	18.3 (5.8)	18.5	7.5
BVMT-R 1-3	24.1 (6.3)	25	9
BVMT-R 25	9.5 (2.1)	10	3
RAVLT 1-5	53.4 (7.6)	53	11
RAVLT 30	11.2 (2.5)	11	4
LM-DR	13.6 (4.2)	14	6
DS-F	9.1 (2.1)	9	2
DS-B	6.7 (1.8)	6	3

*Note.* N = 261. SCD: subjective cognitive decline; SD: standard deviation; IQR: Interquartile Range; BAI: Beck Anxiety Inventory; BNT: Boston Naming Test (30 odd-items version), score = number of errors; BVMT-R 1-3: Brief Visuospatial Memory Test-Revised, sum of trials 1-3; BVMT-R 25: BVMT-R, delayed recall after 25 min; CDT: Clock Drawing Test; DS-B: Digit Span Backward, score; DS-C: Digit Symbol Coding, total score; DS-F: Digit Span Forward, score; GDS-15: Geriatric Depression Scale, 15-item version; LM-DR: Logical Memory Story I, delayed recall; MMSE: Mini-Mental State Examination; P-VF: phonemic verbal fluency (letters N, K, P); RAVLT 1-5: Rey Auditory Verbal Learning Test, sum of trials 1-5; RAVLT 30: Rey Auditory Verbal Learning Test, delayed recall after 30 min; ROCF-C: Rey-Osterrieth Complex Figure, copy; ROCF-R: ROCF, reproduction; S-VF: semantic verbal fluency (A = animals, V = vegetables); TMT: Trail Making Test.

The self-rated QPC was completed by all included participants at baseline (n = 261), whereas the informant-rated version was available at baseline for a subset of 237 individuals. Among participants with available informant-reported baseline questionnaires, 128 (54.0%) informants were the participant's spouse, 67 (28.3%) a son or daughter, and 31 (13.1%) indicated another, unspecified relationship; 11 informants (4.6%) did not report their relationship.

### Pattern of SCCs in self- versus informant reports

Patients reported significantly higher overall SCC scores than informants (M = 4.0, SD = 2.1 versus M = 2.5, SD = 2.2; p < 0.001, d = 0.73), based on analyses restricted to participants with both self- and informant-reported baseline questionnaires. All patient–informant comparisons of complaint prevalence were conducted within this same subset of participants (n = 237). Across all items, the prevalence of complaints was consistently higher in patient reports. This difference was most pronounced for memory-related complaints, *limitations in daily activities*, and *word-finding difficulties*. Among the specific complaints, *word-finding difficulties* were the most frequently reported by both patients (67.8%) and informants (43.0%), followed by *difficulties with recalling past events* (66.3% and 47.3%, respectively). In contrast, *spatial orientation difficulties* were the least frequently reported by both groups (17.6% and 9.7%, respectively). Detailed results are presented in Table 2.

**Table 2.** Prevalence of SCCs reported in the QPC.

	PATIENT (n = 261)		INFORMANT (n = 237)				
	Frequency % (n)				Patient-Informant differences		
	YES	MISSING	YES	MISSING	McNemar χ^2^	*p* ^¥^	OR (95% CI, patients vs. informants)
1. Impression of memory change	55.9 (146)	0.8 (2)	32.5 (77)	0.8 (2)	33.5	<0.001	4.4 (2.6–7.6)
2. Impression of worse memory in comparison to peers	57.1 (149)	0.8 (2)	21.5 (56)	0.4 (1)	59.3	<0.001	8.1 (4.3–15.1)
3. Difficulties with recalling past events	66.3 (173)	1.1 (3)	47.3 (112)	0	30.7	<0.001	5.0 (2.7–9.3)
4. Forgetting about appointments	27.2 (71)	1.5 (4)	21.5 (51)	1.3 (3)	4.9	0.026	2.1 (1.1–3.8)
5. Losing things	29.1 (76)	0.4 (1)	19.4 (46)	1.3 (3)	11.3	0.001	3.3 (1.6–6.7)
6. Spatial orientation difficulties	17.6 (46)	1.1 (3)	9.7 (23)	0	9.3	0.002	3.4 (1.5–7.4)
7. Forgetting about past experiences	26.1 (68)	0.8 (2)	14.3 (34)	0.8 (2)	13.0	<0.001	3.0 (1.6–5.5)
8. Word finding difficulties	67.8 (177)	0.8 (2)	43.0 (102)	0.4 (1)	40.5	<0.001	5.9 (3.2–10.9)
9. Limitation in daily activities	21.5 (56)	0.8 (2)	14.8 (35)	0.8 (2)	3.7	0.053	2.0 (1.0–3.9)
10. Personality change	36.4 (95)	1.1 (3)	23.6 (56)	0	12.2	<0.001	2.8 (1.6–4.9)
	**m (SD)**				**t**	***p* ^¥¥^**	**d (95% CI)**
Total Score	4.0 (2.1)		2.5 (2.2)		11.7	<0.001	0.8 (0.6–0.9)

*Note.* All QPC prevalence data shown in this table refer to baseline assessments. m: mean; SCCs: subjective cognitive complaints; SD standard deviation; p^¥^ values refer to within-pair differences between patient- and informant-reported responses, tested using continuity-corrected McNemar's test (pairwise deletion for missing data); OR refers to odds ratios that were calculated from discordant pairs, defined as (patient = yes, informant = no) versus (patient = no, informant = yes), values of OR > 1 indicate that patients were more likely to endorse the item (e.g., OR = 5, a “yes” was about five times more likely from the patient); p^¥¥^ refers to the main effect between the patient- and informant-reported versions of the QPC using the paired sample t-test; d: Cohen's d.

### Associations of self- versus informant-reported SCCs with longitudinal cognitive decline

Tables 3a-c summarize the results of linear mixed effects models assessing the associations between the QPC-total score and ten specific QPC-items, as reported by patients or their informants, with changes in the global cognitive composite (Table 3a), memory composite (Table 3b), and executive function/processing speed composite (Table 3c). Complete mixed-effects model outputs, including full coefficients for all predictors and model fit statistics, are provided in the Supplemental Material (Supplemental Table 1a-d and Supplemental Table 2).

**Table 3a.** Linear mixed effect models predicting decline in global cognitive composite Z-score as a function of fixed and random effects: the self- and informant-reported specific QPC–items and QPC–total score.

		PATIENT (n = 261)						INFORMANT (n = 237)					
	Global cognitive composite z-score	Item (95% CI)	p	Time variable (95% CI)	p	Item*time variable (95% CI)	p	Item (95% CI)	p	Time variable (95% CI)	p	Item*time variable (95% CI)	p
		Estimates (CI)						Estimates (CI)					
1	Impression of memory change^##, §§^	0.07 (−0.05, 0.19)	0.276	0.02 (−0.01, 0.05)	0.172	**−0.06** (**−0.10, −0.02)**	**0**.**005**	0.28 (0.12, 0.42)	<0.001	0.04 (0.02, 0.07)	<0.001	**−0.14** (**−0.18, −0.09)**	**<0**.001*
2	Impression of worse memory in comparison to peers^#, §§^	0.05 (−0.08, 0.17)	0.473	0.02 (−0.01, 0.04)	0.284	**−0.05** (**−0.09, −0.01)**	**0**.**016**	0.18 (0.03, 0.33)	0.022	0.02 (−0.00, 0.04)	0.051	**−0.14** (**−0.19, −0.09)**	**<0**.001*
3	Difficulties with recalling past events^####, §§§^	0.09 (−0.04, 0.21)	0.171	0.02 (−0.01, 0.05)	0.284	**−0.04** (**−0.08, −0.00)**	**0**.**046**	0.12 (0.00, 0.25)	0.059	0.03 (0.00, 0.05)	0.028	**−0.07** (**−0.12, −0.03)**	**0**.001*
4	Forgetting about appointments^####, §§§§§^	0.08 (−0.06, 0.22)	0.258	0.00 (−0.02, 0.02)	0.863	−0.05 (−0.09, 0.00)	0.058	0.07 (−0.10, 0.24)	0.422	0.01 (−0.01, 0.03)	0.477	−0.05 (−0.11, 0.01)	0.091
5	Losing things^#####, §§§§^	0.12 (−0.01, 0.26)	0.079	0.00 (−0.02, 0.02)	0.977	−0.03 (−0.07, 0.02)	0.208	0.07 (−0.09, 0.23)	0.397	0.01 (−0.01, 0.03)	0.460	−0.04 (−0.09, 0.01)	0.123
6	Spatial orientation difficulties^#######, §§§§§§^	0.04 (−0.12, 0.20)	0.620	−0.00 (−0.02, 0.02)	0.940	−0.04 (−0.10, 0.01)	0.109	0.00 (−0.22, 0.23)	0.991	0.01 (−0.01, 0.03)	0.317	**−0.11** (**−0.18, −0.03)**	**0**.**005**
7	Forgetting about past experiences^#####, §§§§^	0.00 (−0.14, 0.14)	1.00	0.00 (−0.02, 0.02)	0.847	−0.03 (−0.08, 0.02)	0.175	0.13 (−0.05, 0.31)	0.170	0.01 (−0.01, 0.03)	0.329	**−0.07** (**−0.13, −0.02)**	**0**.**014**
8	Word finding difficulties^######, §^	−0.02 (−0.15, 0.11)	0.749	−0.00 (−0.04, 0.03)	0.772	−0.00 (−0.05, 0.04)	0.820	0.07 (−0.05, 0.20)	0.249	0.02 (−0.01, 0.04)	0.193	−0.04 (−0.08, 0.00)	0.061
9	Limitation in daily activities^#####, §§§^	0.09 (−0.07, 0.25)	0.292	0.00 (−0.02, 0.02)	0.829	**−0.08** (**−0.13, −0.03)**	**0**.003*	−0.08 (−0.26, 0.09)	0.347	0.02 (−0.00, 0.04)	0.112	**−0.08** (**−0.14, −0.03)**	**0**.003*
10	Personality change^###, §§^	0.16 (0.04, 0.29)	0.012	0.01 (−0.01, 0.04)	0.264	**−0.07** (**−0.11, −0.02)**	**0**.002*	−0.04 (−0.19, 0.12)	0.646	0.01 (−0.01, 0.03)	0.384	−0.04 (−0.09, 0.01)	0.081
Total score^########, §§§§§§§^		0.02 (−0.01, 0.05)	0.148	0.05 (0.01, 0.09)	0.006	**−0.02** (**−0.03, −0.01)**	**<0**.001*	0.02 (−0.01, 0.05)	0.312	0.05 (0.02, 0.08)	0.001	**−0.02** (**−0.03, −0.01)**	**<0**.001*

*Note.* All models include fixed effects for age, sex, and education; cells shaded in grey and shown in bold indicate statistically significant results exclusively for the item*time interaction; * denotes results that remained statistically significant after Bonferroni correction (p < 0.005); sample sizes shown for patient and informant reports refer to participants with available baseline questionnaires; informant-reported QPC data were additionally included in the linear mixed effects models whenever available across follow-up visits, even if baseline informant-reported questionnaires were missing, in order to maximize the use of available longitudinal data; number of observations in self-reported QPC version: ^#^ 835; ^##^ 844; ^###^845; ^####^846; ^#####^849; ^######^850; ^#######^852; ^########^855; number of observations in informant-reported QPC version: ^§^ 773; ^§§^ 774; ^§§§^ 775; ^§§§§^ 776; ^§§§§§^ 777; ^§§§§§§^ 779; ^§§§§§§§^ 781.

**Table 3b.** Linear mixed effect models predicting decline in memory composite Z-score as a function of fixed and random effects: the self- and informant-reported specific QPC–items and QPC–total score.

		PATIENT (n = 261)						INFORMANT (n = 237)					
	Memory composite z-score	Item (95% CI)	P (95% CI)	Time variable (95% CI)	p	Item*time variable (95% CI)	p	Item (95% CI)	p	Time variable (95% CI)	p	Item*time variable (95% CI)	p
		Estimates (CI)						Estimates (CI)					
1	Impression of memory change^##, §§^	0.05 (−0.12, 0.22)	0.589	0.10 (0.06, 0.14)	<0.001	**−0.07** (**−0.13, −0.01)**	**0**.**014**	0.41 (0.21, 0.61)	<0.001	0.13 (0.10, 0.17)	<0.001	**−0.17** (**−0.23, −0.10)**	**<0**.001*
2	Impression of worse memory in comparison to peers^#, §§^	0.01 (−0.17,0.19)	0.946	0.10 (0.06, 0.14)	<0.001	**−0.06** (**−0.12, −0.00)**	**0**.**039**	0.19 (−0.03, 0.42)	0.088	0.10 (0.07, 0.14)	<0.001	**−0.15** (**−0.23, −0.07)**	**<0**.001*
3	Difficulties with recalling past events^####, §§§^	0.07 (−0.11, 0.25)	0.471	0.10 (0.06, 0.14)	<0.001	−0.05 (−0.11, 0.00)	0.072	0.12 (−0.07, 0.31)	0.206	0.10 (0.07, 0.14)	<0.001	−0.06 (−0.12, 0.01)	0.082
4	Forgetting about appointments^####, §§§§§^	0.17 (−0.03, 0.37)	0.096	0.08 (0.05, 0.12)	<0.001	−0.06 (−0.12, 0.01)	0.101	0.15 (−0.09, 0.39)	0.216	0.09 (0.06, 0.12)	<0.001	−0.08 (−0.16, 0.01)	0.078
5	Losing things^#####, §§§§^	0.30 (0.10, 0.50)	0.003	0.09 (0.06, 0.12)	<0.001	**−0.07** (**−0.14, −0.01)**	**0**.**03**	0.24 (0.00, 0.47)	0.049	0.10 (0.07, 0.13)	<0.001	**−0.10** (**−0.18, −0.03)**	**0**.**006**
6	Spatial orientation difficulties^#######, §§§§§§^	0.15 (−0.08, 0.38)	0.206	0.08 (0.05, 0.11)	<0.001	−0.06 (−0.13, 0.02)	0.134	−0.17 (−0.50, 0.16)	0.298	0.09 (0.06, 0.12)	<0.001	−0.06 (−0.16, 0.05)	0.307
7	Forgetting about past experiences^#####, §§§§^	−0.02 (−0.22, 0.19)	0.877	0.08 (0.05, 0.11)	<0.001	−0.04 (−0.11, 0.03)	0.310	0.17 (−0.09, 0.44)	0.191	0.10 (0.07, 0.13)	<0.001	**−0.13** (**−0.21, −0.04)**	**0**.003*
8	Word finding difficulties^######, §^	−0.07 (−0.26, 0.11)	0.446	0.06 (0.02, 0.11)	0.008	0.01 (−0.05, 0.07)	0.711	0.11 (−0.07, 0.30)	0.238	0.10 (0.06, 0.13)	<0.001	−0.03 (−0.09, 0.02)	0.263
9	Limitation in daily activities^#####, §§§^	0.05 (−0.18, 0.28)	0.692	0.08 (0.05, 0.11)	<0.001	−0.07 (−0.15, 0.00)	0.064	−0.02 (−0.27, 0.24)	0.899	0.10 (0.07, 0.13)	<0.001	**−0.11** (**−0.19, −0.03)**	**0**.**006**
10	Personality change^###, §§^	0.19 (0.00, 0.37)	0.049	0.09 (0.06, 0.12)	<0.001	−0.06 (−0.12, 0.00)	0.052	−0.08 (−0.31, 0.15)	0.489	0.09 (0.05, −0.12)	<0.001	−0.02 (−0.10, 0.05)	0.548
Total score^########, §§§§§§§^		0.03 (−0.01, 0.07)	0.169	0.14 (0.09, 0.20)	<0.001	**−0.02** **(−0.03, −0.01)**	**0**.001*	0.03 (−0.01, 0.07)	0.176	0.14 (0.10, 0.18)	<0.001	**−0.03** (**−0.04, −0.01)**	**<0**.001*

*Note.* All models include fixed effects for age, sex, and education; cells shaded in grey and shown in bold indicate statistically significant results exclusively for the item*time interaction; * denotes results that remained statistically significant after Bonferroni correction (p < 0.005); sample sizes shown for patient and informant reports refer to participants with available baseline questionnaires; informant-reported QPC data were additionally included in the linear mixed effects models whenever available across follow-up visits, even if baseline informant-reported questionnaires were missing, in order to maximize the use of available longitudinal data; number of observations in self-reported QPC version: ^#^ 834; ^##^ 843; ^###^844; ^####^845; ^#####^848; ^######^849; ^#######^851; ^########^854; number of observations in informant-reported QPC version: ^§^ 772; ^§§^ 773; ^§§§^ 774; ^§§§§^ 775; ^§§§§§^ 776; ^§§§§§§^ 778; ^§§§§§§§^ 780.

**Table 3c.** Linear mixed effect models predicting decline in executive function/processing speed composite Z-score as a function of fixed and random effects: the self- and informant-reported specific QPC–items and QPC–total score.

			PATIENT (n = 261)						INFORMANT (n = 237)				
	Executive—processing speed composite z-score	Item(95% CI)	p	Time variable (95% CI)	p	Item*time variable (95% CI)	p	Item (95% CI)	p	Time variable (95% CI)	p	Item*time variable (95% CI)	p
		Estimates (CI)						Estimates (CI)					
1	Impression of memory change ^##, §§^	0.05 (−0.07, 0.17)	0.376	−0.05 (−0.08, −0.02)	<0.001	**−0.04** (**−0.08, −0.00)**	**0**.**043**	0.15 (0.01, 0.28)	0.034	−0.04 (−0.06, −0.02)	0.001	**−0.09** (**−0.13, −0.04)**	**<0**.001*
2	Impression of worse memory in comparison to peers^#, §§^	0.02 (−0.11, 0.14)	0.808	−0.05 (−0.08, −0.03)	<0.001	−0.04 (−0.07, 0.00)	0.075	0.05 (−0.11, 0.20)	0.531	−0.06 (−0.08, −0.03)	<0.001	**−0.08** (**−0.13, −0.03)**	**0**.003*
3	Difficulties with recalling past events^####, §§§^	0.05 (−0.08, 0.17)	0.482	−0.06 (−0.09, −0.03)	<0.001	−0.02 (−0.06, 0.02)	0.340	0.04 (−0.08, 0.17)	0.507	−0.05 (−0.08, −0.02)	<0.001	**−0.05** (**−0.10, −0.01)**	**0**.**014**
4	Forgetting about appointments^####, §§§§§§^	−0.01 (−0.15, 0.13)	0.868	−0.07 (0.09, −0.05)	<0.001	−0.02 (−0.07, 0.02)	0.335	−0.05 (−0.22, 0.11)	0.525	−0.07 (−0.09, −0.05)	<0.001	−0.01 (−0.07, 0.06)	0.855
5	Losing things^#####, §§§§§^	−0.08 (−0.22, 0.06)	0.252	−0.08 (−0.10, −0.05)	<0.001	0.01 (−0.03, 0.06)	0.533	−0.01 (−0.18, 0.15)	0.863	−0.07 (−0.09, −0.05)	<0.001	0.01 (−0.04, 0.06)	0.726
6	Spatial orientation difficulties^#######, §§§§§§§^	−0.09 (−0.26, 0.07)	0.265	−0.07 (−0.09, −0.05)	<0.001	−0.01 (−0.06, 0.05)	0.784	−0.10 (−0.33, 0.13)	0.396	−0.06 (−0.08, −0.04)	<0.001	−0.07 (−0.15, 0.00)	0.054
7	Forgetting about past experiences^#####, §§§§§^	−0.05 (−0.19, 0.10)	0.514	−0.07 (−0.09, −0.05)	<0.001	−0.01 (−0.06, 0.04)	0.769	−0.03 (−0.21, 0.15)	0.734	−0.07 (−0.09, −0.04)	<0.001	0.00 (−0.06, 0.06)	0.922
8	Word finding difficulties^######, §^	0.03 (−0.10, 0.16)	0.642	−0.06 (−0.09, −0.03)	<0.001	−0.02 (−0.06, 0.02)	0.385	0.03 (−0.10, 0.16)	0.635	−0.05 (−0.08, −0.02)	<0.001	−0.04 (−0.08, 0.00)	0.062
9	Limitation in daily activities^#####, §§§^	0.05 (−0.11, 0.21)	0.548	−0.06 (−0.09, −0.04)	<0.001	**−0.05** (**−0.11, −0.00)**	**0**.**048**	−0.06 (−0.24, 0.12)	0.526	−0.06 (−0.08, −0.04)	<0.001	−0.05 (−0.10, 0.01)	0.103
10	Personality change^###, §§^	0.04 (−0.09, 0.17)	0.526	−0.06 (−0.08, −0.04)	<0.001	**−0.04** (**−0.09, −0.00)**	**0**.**041**	0.01 (−0.14, 0.17)	0.859	−0.05 (−0.08, −0.03)	<0.001	**−0.06** (**−0.11, −0.01)**	**0**.**019**
Total score^########, §§§§§§§§^		−0.01 (−0.04, 0.02)	0.534	−0.05 (−0.08, −0.01)	0.015	**−0.01** **(−0.02, −0.00)**	**0.044**	−0.01 (−0.04, 0.02)	0.513	−0.04 (−0.07, −0.01)	0.004	**−0.01** (**−0.02, −0.00)**	**0**.004*

*Note.* All models include fixed effects for age, sex, and education; cells shaded in grey and shown in bold indicate statistically significant results exclusively for the item*time interaction; * denotes results that remained statistically significant after Bonferroni correction (p < 0.005); sample sizes shown for patient and informant reports refer to participants with available baseline questionnaires; informant-reported QPC data were additionally included in the linear mixed effects models whenever available across follow-up visits, even if baseline informant-reported questionnaires were missing, in order to maximize the use of available longitudinal data; number of observations in self-reported QPC version: ^#^ 835; ^##^ 844; ^###^845; ^####^846; ^#####^849; ^######^850; ^#######^852; ^########^855; number of observations in informant-reported QPC version: ^§^ 773; ^§§^ 774; ^§§§^ 775; ^§§§§^ 775; ^§§§§§^ 776; ^§§§§§§^ 777; ^§§§§§§§^ 779; ^§§§§§§§§^ 781.

The tables report three key effects: 1. The impact of the QPC-item or QPC-total score on baseline cognitive performance, where a significant negative effect indicates lower baseline performance for a “Yes” response or higher total score. 2. The effect of time on cognitive performance, with a significant negative effect indicating decline in performance over time. 3. The interaction between time and QPC-item or QPC-total score, which was the primary outcome of interest. A significant negative interaction indicates that participants endorsing the QPC-item or with higher QPC-total scores experienced a greater cognitive decline over time, on average.

Several QPC-items and the QPC-total score were related to longitudinal cognitive decline in each of the domains; in contrast, very few specific QPC-items, and not the QPC-total score, were associated with baseline cognitive performance.

### Rate of change in global cognition

A higher QPC-total score was significantly associated with decline in the global cognitive composite, with a comparable magnitude of effects observed for both patient and informant endorsements.

When examining individual QPC-items, informant-reported *impression of memory change* and *impression of worse memory in comparison to peers* were associated with steeper decline in global cognition than the QPC-total score and remained significant after Bonferroni correction (Table 3a).

Several other QPC-items were associated with global cognitive decline at the nominal significance level but did not consistently remain significant after correction. These included other memory-related complaints (*difficulties with recalling past events* and *forgetting about past experiences*) as well as non-memory complaints such as *spatial orientation difficulties*, *limitations in daily activities*, and *personality change*. On average, the magnitude of effects tended to be larger for informant reports than for self-reports.

*Spatial orientation difficulties* were significantly associated with greater global cognitive decline only when endorsed by informants, although the effect was right at the significance threshold (p = 0.005). Conversely, *personality change* was significantly associated with greater global cognitive decline solely in patient reports. The estimated interaction effects between subjective cognitive complaints and time on global cognitive composite scores are illustrated in Figure 1.

**Figure 1.**
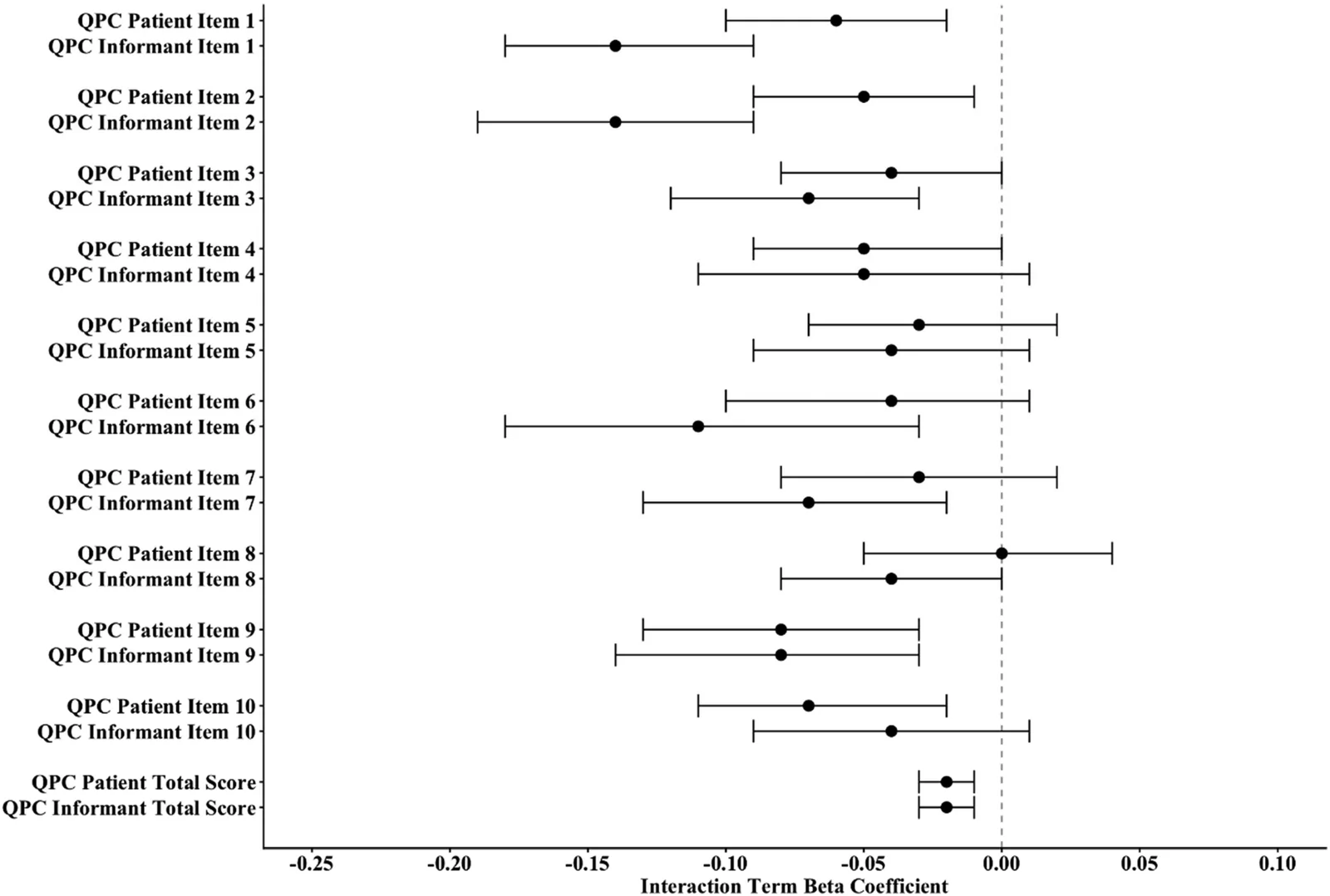
Associations between subjective cognitive complaints and longitudinal change in global cognitive composite scores.

*Note.* Points represent unstandardized regression coefficients for the QPC × time interaction terms derived from linear mixed-effects models predicting longitudinal change in the global cognitive composite score; horizontal lines indicate 95% confidence intervals. Results are shown separately for patient- and informant-reported QPC total scores and individual QPC items.

Item key names: Item 1 = Impression of memory change; Item 2 = Impression of worse memory in comparison to peers; Item 3 = Difficulties with recalling past events; Item 4 = Forgetting about appointments; Item 5 = Losing things; Item 6 = Spatial orientation difficulties; Item 7 = Forgetting about past experiences; Item 8 = Word finding difficulties; Item 9 = Limitation in daily activities; Item 10 = Personality change.

### Rate of change in memory

A higher QPC-total score was significantly associated with decline in the memory composite, with a comparable magnitude of effects observed for both patient and informant endorsements.

When examining individual QPC-items, informant-reported *impression of memory change* and *impression of worse memory in comparison to peers* were associated with steeper memory decline than the QPC-total score. These associations remained statistically significant after Bonferroni correction (Table 3b), consistent with the pattern observed for the global cognitive composite.

Several other QPC-items showed associations with memory decline at the nominal significance level, including *forgetting about past experiences* as well as non-memory complaints such as *losing things* and *limitations in daily activities*. After Bonferroni correction, only informant-reported *forgetting about past experiences* remained statistically significant. On average, the magnitude of effects tended to be larger for informant reports than for self-reports; however, confidence intervals overlapped in most cases.

### Rate of change in executive function/processing speed

A higher QPC-total score was significantly associated with decline in the executive function/processing speed composite, with similar effects observed for both patient and informant endorsements, consistent with findings for the global cognitive and memory composites. After Bonferroni correction, only informant-reported QPC-total score remained statistically significant.

At the item-level, compared to the QPC-total score, informant-reported *impression of memory change* and *impression of worse memory in comparison to peers* were associated with a steeper decline in executive function/processing speed, and both associations remained statistically significant after Bonferroni correction.

Several other QPC-items showed associations with executive function/processing speed decline at the nominal significance level, including *difficulties with recalling past events*, *limitations in daily activities*, and *personality change;* however, none of these associations remained statistically significant after correction.

Sensitivity analyses in which the sample was further restricted to participants with both patient- and informant-reported baseline questionnaires yielded comparable patterns of results across global cognition, memory, and executive function/processing speed (see Supplemental Table 3a–c).

An exploratory supplementary analysis indicated that the magnitude of patient-informant discrepancies in QPC total scores did not differ systematically by informant relationship.

## Discussion

In this longitudinal study of a clinical sample of older adults with SCD, we examined the associations between self- and informant-reported SCCs and cognitive decline in global cognition, memory, and executive function/processing speed, which are cognitive composites considered most sensitive to subtle but clinically meaningful changes on the cognitive trajectories of pathological aging.^30^

Using the 10-item QPC, higher QPC-total score was associated with cognitive worsening across all three cognitive composites, with similar effects for both patient and informant reports. At the item level, a subset of specific complaints showed more pronounced and consistent associations with longitudinal cognitive decline than the QPC-total score, in line with our hypothesis. These were primarily memory-related, most notably *impression of memory change* and *impression of worse memory in comparison to peers,* particularly when reported by informants. Other memory- and non-memory-related complaints showed associations in the same direction but were less consistent after conservative correction for multiple comparisons. Informants generally reported fewer complaints than patients themselves; however, as expected, their reports were often more strongly associated with worsening cognitive performance, even within the SCD group, where anosognosia is unlikely to occur. An exception was *personality change*, which emerged as a significant marker of cognitive decline, particularly when reported by patients themselves.

Together, these findings suggest that specific SCCs, rather than a global score, may provide greater specificity in capturing longitudinal progressive cognitive decline.^23,31^ While most studies assessing the diagnostic and predictive value of SCCs rely on a single global score,^32,33^ emerging evidence suggests that as cognitive complaints become increasingly common with age, it is their specific pattern, rather than their total number, that more accurately reflects an underlying pathological process.^8,9,34^ Consistent with these findings, our previous cross-sectional research in older adults from both community- and a clinically-based cohorts,^11,12^ which also utilized the QPC questionnaire, showed that while a higher overall QPC score was most closely associated with greater depressive symptoms, specific individual complaints more accurately reflected poorer cognitive performance.

One possible explanation for the greater informative value of specific complaints compared with the QPC-total score is that the relationship between the overall level of SCCs and objective cognitive decline may not be linear.^1,4,10^ At higher levels of complaint burden, global scores may increasingly reflect a broader range of influences, including affective and other neuropsychiatric symptoms, which are known to influence subjective reporting^2,11^ and co-occur with early neurodegenerative changes.^35,36^ In contrast, when the overall level of subjective complaints is relatively low or moderate, affirmative responses to specific SCCs items may be more specifically linked to subtle cognitive changes and early cognitive decline risk than a global complaint score.^8,11,12,14,37^

Within this framework, memory-related SCCs appear particularly relevant. Previous community-based studies have consistently reported that memory-related subjective cognitive complaints are linked to subsequent cognitive decline and an elevated risk of developing MCI or dementia.^14,22,23,31^ Additionally, a recent cross-sectional study found that self-reported memory complaints and seeking medical evaluation for memory concerns were associated with elevated amyloid levels on positron emission tomography imaging.^8^ In line with this literature, our findings suggest that specific episodic memory complaints may reflect subtle changes in objective cognitive performance in individuals with SCD, consistent with the central role of episodic memory in early cognitive alterations associated with pathological aging.^38–40^

The current findings further extend this notion by showing that not all memory-related complaints are equally informative, and that two items referencing 1) perceived change (*impression of memory change*) and 2) comparison with age-matched peers (*impression of worse memory in comparison to peers)* are especially relevant for longitudinal cognitive decline, particularly when reported by informants. These two items showed consistent associations with decline across global cognition, memory, and executive function/processing speed composites, and a steeper cognitive decline than that estimated by the QPC-total score. These results validate and extend our previous cross-sectional findings, in which a self-reported *impression of worse memory in comparison to peers* was linked to poorer cognitive performance in both a cognitively normal community-based sample and a clinic-based sample of non-demented older adults.^11,12^ Importantly, this pattern converges with prior studies indicating that complaints explicitly framed in relation to age-matched peers show particularly strong and consistent associations with objective cognitive performance.^37,41^

Regarding the *impression of memory change*, the present findings contrast with our earlier work,^11,12^ where this complaint appeared non-specific to pathological aging. This was likely due to its high prevalence among cognitively normal older adults from the community, as well as to reduced insight in patients with MCI. However, another previous cross-sectional study from the community^13^ showed that acknowledgement of progressive memory change differentiated cognitively normal older individuals with high amyloid-β load, indicative of preclinical AD, from those with low burden. Furthermore, acknowledgement of memory worsening associated with worry was found to be a stronger risk factor for conversion to dementia compared to memory worsening without worry.^10^ Together, the present findings and previous research suggest that the clinical relevance of *impression of memory change* depends on the context and population studied: it represents a high-risk marker when it reflects progressive decline and is reported by worried, medical help seeking individuals.

While memory-related complaints are known indicators of pathological aging, recent research suggests that non-memory complaints may also be relevant.^31,34,42^ Several non-memory SCCs assessed in the QPC emerged as promising indicators of impending cognitive impairment. In our earlier cross-sectional research,^11,12^ self-reported *spatial orientation difficulties*, *limitations in daily activities*, and *personality change* were already linked to lower cognitive performance. Extending these findings, the present longitudinal study demonstrates that, in SCD, these complaints are associated with steeper cognitive decline, particularly when endorsed by informants, with the exception of *personality change*, which remained statistically significant after Bonferroni correction only in self-report. The emergence of these non-memory complaints may reflect hippocampal dysfunction manifesting through non-memory features, such as spatial navigation difficulties.^42^ Additionally, even at the SCD stage, neurodegeneration may extend to non-memory brain regions, particularly the parietal lobe, contributing to these complaints.^6^

In the current study, *spatial orientation difficulties* were associated with a steeper decline in global cognition, but only when reported by informants. This finding aligns with our recent cross-sectional study, in which only informant-reported navigation difficulties were linked to poorer navigation performance, greater AD-related atrophy, and increased Aβ positivity.^42^ In that study, the effect was primarily observed in aMCI patients, with no significant association found in those with SCD, likely due to limited statistical power resulting from a small sample size.^42^ The larger sample and longitudinal design of the present study provide further support for the utility of informant-reported spatial orientation complaints as a potential early marker of pathological cognitive decline, even among patients with SCD. This aligns with prior evidence that objective spatial orientation deficit is common in the prodromal stage, and may even be detectable in preclinical stages of AD,^15,43,44^ and that such difficulties may also manifest as specific SCCs.^42,45^

*Limitations in daily activities* were another non-memory complaint associated with longitudinal decline when endorsed by informants. This is particularly notable given that the sample consisted of cognitively unimpaired individuals with SCD, who are, by definition, fully independent in daily functioning.^46,47^ However, it is important to consider that the phrasing of *Limitations in daily activities* in the QPC specifies that these limitations arise from fear of making mistakes. While this concern may reflect an individual's awareness of declining functional abilities, it could also be associated with anxiety or depressive symptoms.

Another non-memory complaint associated with longitudinal cognitive decline, when reported by patients, was *personality change*. In our previous cross-sectional study, this QPC-item was more closely linked to higher depressive symptoms than to poor cognitive performance,^11^ whereas our current findings support its relevance as an early indicator of cognitive worsening. This is consistent with current models of neuropsychiatric symptoms, which propose that both depressive and cognitive changes may represent direct consequences of early neurodegenerative processes.^35,36^ Notably, *personality change* was the only complaint for which the self-report, but not the informant-report, significantly predicted cognitive deterioration. Although informant-reported complaints generally showed stronger and more consistent associations with longitudinal cognitive decline, consistent with prior research,^19,32,48,49^ our findings highlight that self-reported concerns in SCD also provide valuable insights. This suggests that patients and informants may capture distinct aspects of pathological decline.^8,23,24^ Specifically, in the case of *personality change,* the features specified in the QPC—including turning inward, reducing social contact, or losing interest in activities—likely reflect internalized symptoms that may remain less apparent to others, much like depressive or anxious experiences.^50^

*Word-finding difficulties* are a common complaint in older adults.^11,12^ They were also among the most frequently reported QPC items by both patients and informants in the current study, where no associations between this complaint and longitudinal cognitive decline in individuals with SCD were found. This aligns with our previous cross-sectional findings, where *word-finding difficulties* were not linked to poorer cognitive performance in either cognitively normal individuals from the community or non-demented patients from a memory clinic.^11,12^ While word-finding problems related to AD are often attributed to degradation of semantic knowledge,^16,51^ in normal aging they may stem from difficulties in accessing lexical word forms.^52^ These two potential underlying mechanisms may explain why this complaint lacks specificity for pathological aging. Supporting this interpretation, a recent longitudinal study in cognitively normal older adults from the community found that impaired understanding of spoken instructions predicted progression to MCI or dementia, whereas simple word-finding difficulties did not.^31^

Taken together, only some of the QPC-items hold longitudinal predictive value in SCD. Furthermore, the clinical relevance of the overall, rather small, magnitude of statistically significant effects could be questioned as well as the statistical significance of items that did not reach the Bonferroni cut-off. However, these findings should be interpreted in an appropriate context. Importantly, the analysis included only individuals with SCD who, by definition, perform within normal limits on neuropsychological tests, and numerous models were necessarily conducted to elucidate the potential mechanisms behind individual endorsements of items on the QPC and cognitive decline. Moreover, the effect sizes observed in this study were comparable to those reported in previous community-based longitudinal studies that also examined associations between SCCs and cognitive decline.^23,34^ Another important consideration is that the magnitude of detected cognitive changes may be reduced due to test-retest effects.^53^ Cognitively normal individuals, particularly those who are high-functioning and derive minimal benefit from repeated assessments, are at increased risk for future cognitive impairment and underlying neurodegeneration. Thus, even subtle decline in cognitive performance may carry meaningful clinical implications within this study population.

To the best of our knowledge, this is among the first longitudinal studies in a clinically ascertained SCD sample directly comparing the distinct perspectives of patient- and informant-reported SCCs. The longitudinal design, with multiple follow-up visits allowed for a robust investigation of cognitive trajectories over an extended period. In addition, we used composite scores for memory, executive function/processing speed, and global cognition, which can detect subtle performance decline more reliably and effectively than a single cognitive test.^30^

Though our study has several strengths, certain limitations should be acknowledged. We examined associations between SCCs and long-term cognitive decline, but did not assess the risk of incident MCI or dementia. While dementia is a clinically significant endpoint, reliably determining the predictive value of SCCs for its development would require a longer follow-up. Unlike dementia, where loss of independence defines progression, the distinction between MCI and SCD is less reliable, as it is based solely on test performance rather than functional ability.^54^ The lower diagnostic certainty and continuum-based nature of this distinction may contribute to back-reversion from MCI to SCD.^1^ Thus, focusing on cognitive trajectories rather than rigid syndromological classifications offers a more robust approach to understanding long-term cognitive outcomes in these early stages. In this context, it should be noted that the QPC items *limitation in daily activities* and *personality change*, although significantly associated with cognitive decline over time, are not exclusively cognitive in nature and may reflect overlapping cognitive, functional, or neuropsychiatric processes. Additionally, as our sample was drawn from memory clinics, the findings are primarily generalizable to clinical populations. Cultural and healthcare-system differences may influence how SCCs are perceived or reported,^1^ and should therefore be considered when applying these results across culturally and healthcare-diverse contexts.

Future research should incorporate biomarker data to clarify the pathology underlying specific SCC patterns and include larger, more diverse cohorts with longer follow-up to better capture progression from SCD to dementia. In addition, as dyadic reporting gains increasing recognition,^18^ examining the interaction between self- and informant-reports over time, from preclinical to prodromal stages, may further enhance understanding of the clinical relevance of SCCs. Future studies with larger samples and longitudinal designs may further help clarify whether informant characteristics, including the nature of the patient-informant relationship, contribute to variability in informant-reporting relative to patient self-report, as suggested by prior work.^55^

### Conclusion

In conclusion, this study adds novel longitudinal evidence on the clinical relevance of specific self- and informant-reported cognitive complaints in SCD population. Using a longitudinal, clinically based design, we show that specific SCCs, particularly those reported by informants, are associated with more pronounced longitudinal cognitive decline than a global SCC score. Both memory- and non-memory-related complaints were significantly associated with worsening cognition over time, emphasizing the importance of shifting focus from the quantity to the specific nature of cognitive complaints.

Although clinicians at early stages like SCD often rely primarily on patient self-reports, our findings underscore the valuable contribution of informant observations to early diagnostic evaluations. These results expand upon our earlier work^11,12^ and support the integration of structured SCC assessments, such as the QPC, with its brief binary-response format, as a practical tool for improving early clinical evaluation of cognitive difficulties. Given its structure, the QPC is well suited for clinical use and offers potential for digital adaptation to enable remote cognitive monitoring.

## Supplemental Material

sj-docx-1-alz-10.1177_13872877261443656 - Supplemental material for Evaluating self- and informant-reported complaints as predictors of cognitive progression in subjective cognitive decline: A clinically-based evaluation Supplemental material, sj-docx-1-alz-10.1177_13872877261443656 for Evaluating self- and informant-reported complaints as predictors of cognitive progression in subjective cognitive decline: A clinically-based evaluation by Hana Horakova, Dylan J. Jester, Terezie Vohradkova, Katerina Sheardova, Jan Ondrej, Veronika Matuskova, Adela Fendrych Mazancova, Tomas Nikolai, Ross Andel, Ondrej Lerch, Jan Laczó, Jakub Hort and Martin Vyhnalek in Journal of Alzheimer's Disease
