# Supplementary material for: Evaluating self- and informant-reported complaints as predictors of cognitive progression in subjective cognitive decline: A clinically-based evaluation
Source: J Alzheimers Dis. 2026 Apr 23;111(4):1547–62. doi: 10.1177/13872877261443656 (PMC13627773; doi:10.1177/13872877261443656)
Supplement: Supplementary file 1 [file sj-docx-1-alz-10.1177_13872877261443656.docx]

**Supplemental Material**

**Evaluating self- and informant-reported complaints as predictors of cognitive progression in subjective cognitive decline: A clinically-based evaluation**

| **Supplementary Table 1a**  *Full linear mixed-effects model coefficients for QPC-total score models predicting composite cognitive outcomes.* | | | | | | | |  |  |
| --- | --- | --- | --- | --- | --- | --- | --- | --- | --- |
|  | **Patient** | | | | **Informant** | | | | |
| *Predictors* | *Estimates* | *95% CI* | *p* | | *Estimates* | *95% CI* | *p* | | |
|  | **Global Cognitive Composite Z-Score** | | | | | | | | |
| (Intercept) | 1.75 | (0.99, 2.51) | <0.001 | | 1.77 | (0.99, 2.54) | <0.001 | | |
| Baseline age | -0.05 | (-0.05, -0.04) | <0.001 | | -0.04 | (-0.05, -0.04) | <0.001 | | |
| Sex | 0.31 | (0.17, 0.44) | <0.001 | | 0.26 | (0.13, 0.40) | <0.001 | | |
| Education | 0.05 | (0.03, 0.07) | <0.001 | | 0.05 | (0.03, 0.07) | <0.001 | | |
| QPC-total score | 0.02 | (-0.01, 0.05) | 0.148 | | 0.02 | (-0.01, 0.05) | 0.312 | | |
| Time | 0.05 | (0.01, 0.09) | 0.006 | | 0.05 | (0.02, 0.08) | 0.001 | | |
| QPC-total score × time | -0.02 | (-0.03, -0.01) | <0.001 | | -0.02 | (-0.03, -0.01) | <0.001 | | |
| **Random Effects** | σ^2^ = 0.13, τ00 = 0.21, ICC = 0.62, Marginal R^2^ / Conditional R^2^ = 0.318 / 0.740 | | | σ^2^ = 0.12, τ00 = 0.20, ICC = 0.62, Marginal R^2^ / Conditional R^2^ = 0.336 / 0.749 | | | | |  |
|  | **Memory Composite Z-Score** | | | | | | | | |
| (Intercept) | 0.88 | (-0.26, 2.02) | 0.131 | | 0.85 | (-0.34, 2.03) | 0.160 | | |
| Baseline age | -0.04 | (-0.05, -0.03) | <0.001 | | -0.04 | (-0.05, -0.02) | <0.001 | | |
| Sex | 0.48 | (0.27, 0.68) | <0.001 | | 0.43 | (0.22, 0.64) | <0.001 | | |
| Education | 0.05 | (0.02, 0.08) | 0.003 | | 0.05 | (0.02, 0.09) | 0.002 | | |
| QPC-total score | 0.03 | (-0.01, 0.07) | 0.169 | | 0.03 | (-0.01, 0.07) | 0.176 | | |
| Time | 0.14 | (0.09, 0.20) | <0.001 | | 0.14 | (0.10, 0.18) | <0.001 | | |
| QPC-total score × time | -0.02 | (-0.03, -0.01) | 0.001 | | -0.03 | (-0.04, -0.01) | <0.001 | | |
| **Random Effects** | σ^2^ = 0.26, τ00 = 0.47, ICC = 0.64, Marginal R^2^ / Conditional R^2^ = 0.318 / 0.740 | | | σ^2^ = 0.26, τ00 = 0.47, ICC = 0.65, Marginal R^2^ / Conditional R^2^ = 0.186 / 0.713 | | | | |  |
|  | **Executive Function/Processing Speed Composite Z-Score** | | | | | | | | |
| (Intercept) | 2.74 | (1.90, 3.57) | <0.001 | | 2.73 | (1.89, 3.58) | <0.001 | | |
| Baseline age | -0.05 | (-0.06, -0.04) | <0.001 | | -0.05 | (-0.06, -0.04) | <0.001 | | |
| Sex | 0.21 | (0.06, 0.36) | 0.005 | | 0.18 | (0.03, 0.33) | 0.021 | | |
| Education | 0.04 | (0.02, 0.07) | <0.001 | | 0.04 | (0.02, 0.07) | 0.001 | | |
| QPC-total score | -0.01 | (-0.04, 0.02) | 0.534 | | -0.01 | (-0.04, 0.02) | 0.513 | | |
| Time | -0.05 | (-0.08, -0.01) | 0.015 | | -0.04 | (-0.07, -0.01) | 0.004 | | |
| QPC-total score × time variable | -0.01 | (-0.02, -0.00) | 0.044 | | -0.01 | (-0.02, -0.00) | 0.004 | | |
| **Random Effects** | σ^2^ = 0.13, τ00 = 0.26, ICC = 0.67, Marginal R^2^ / Conditional R^2^ = 0.339 / 0.783 | | | σ^2^ = 0.12, τ00 = 0.25, ICC = 0.67, Marginal R^2^ / Conditional R^2^ = 0.352 / 0.787 | | | | |  |
| **Note.** All models include fixed effects for age (baseline), sex, and education, as well as time, QPC total score, and the QPC total score × time interaction, and a random intercept for participant. Random effects are summarized per model as participant-level random intercept variance (τ00), residual variance (σ²), and intraclass correlation coefficient (ICC). Marginal and conditional R² are reported for completeness. Models were estimated using all available observations for each participant in order to maximize the use of available longitudinal data. | | | | | | | |  |  |

| **Supplementary Table 1b**  *Full linear mixed-effects model coefficients for QPC-item level models predicting global cognitive composite score.* | | | | | | | | | |
| --- | --- | --- | --- | --- | --- | --- | --- | --- | --- |
|  | **Patient** | | | | **Informant** | | | |  |
| *Predictors* | *Estimates* | *95% CI* | *p* | *Estimates* | | *95% CI* | *p* |  |  |
|  | 1. Impression of memory change | | | | | | | |  |
| (Intercept) | 1.76 | (1.00, 2.52) | <0.001 | 1.63 | | (0.84, 2.41) | <0.001 |  |  |
| Baseline age | -0.05 | (-0.05, -0.04) | <0.001 | -0.04 | | (-0.05, -0.04) | <0.001 |  |  |
| Sex | 0.31 | (0.17, 0.45) | <0.001 | 0.3 | | (0.16, 0.44) | <0.001 |  |  |
| Education | 0.05 | (0.03, 0.07) | <0.001 | 0.05 | | (0.03, 0.07) | <0.001 |  |  |
| QPC-item 1 | 0.07 | (-0.05, 0.19) | 0.276 | 0.28 | | (0.15, 0.42) | <0.001 |  |  |
| Time | 0.02 | (-0.01, 0.05) | 0.172 | 0.04 | | (0.02, 0.07) | <0.001 |  |  |
| QPC-item 1 × time | -0.06 | (-0.10, -0.02) | 0.005 | -0.14 | | (-0.18, -0.09) | <0.001 |  |  |
| **Random Effects** | σ^2^ = 0.13, τ00 = 0.21, ICC = 0.62, Marginal R^2^ / Conditional R^2^ = 0.312 / 0.737 | | | σ^2^ = 0.12, τ00 = 0.21, ICC = 0.63, Marginal R^2^ / Conditional R^2^ = 0.325 / 0.753 | | | |  |  |
|  | 2. Impression of worse memory in comparison to peers | | | | | | | |  |
| (Intercept) | 1.79 | (1.02, 2.55) | <0.001 | 1.81 | | (1.04, 2.58) | <0.001 |  |  |
| Baseline age | -0.05 | (-0.05, -0.04) | <0.001 | -0.05 | | (-0.05, -0.04) | <0.001 |  |  |
| Sex | 0.31 | (0.17, 0.44) | <0.001 | 0.28 | | (0.14, 0.41) | <0.001 |  |  |
| Education | 0.05 | (0.03, 0.07) | <0.001 | 0.05 | | (0.03, 0.07) | <0.001 |  |  |
| QPC-item 2 | 0.05 | (-0.08, 0.17) | 0.473 | 0.18 | | (0.03, 0.33) | 0.022 |  |  |
| Time | 0.02 | (-0.01, 0.04) | 0.284 | 0.02 | | (-0.00, 0.04) | 0.051 |  |  |
| QPC-item 2 × time | -0.05 | (-0.09, -0.01) | 0.016 | -0.14 | | (-0.19, -0.09) | <0.001 |  |  |
| **Random Effects** | σ^2^ = 0.13, τ00 = 0.20, ICC = 0.61, Marginal R^2^ / Conditional R^2^ = 0.315 / 0.730 | | | σ^2^ = 0.12, τ00 = 0.20, ICC = 0.62, Marginal R^2^ / Conditional R^2^ = 0.325 / 0.740 | | | |  |  |
|  | 3. Difficulties with recalling past events | | | | | | | |  |
| (Intercept) | 1.76 | (0.99, 2.53) | <0.001 | 1.72 | | (0.93, 2.51) | <0.001 |  |  |
| Baseline age | -0.05 | (-0.05, -0.04) | <0.001 | -0.04 | | (-0.05, -0.04) | <0.001 |  |  |
| Sex | 0.31 | (0.17, 0.44) | <0.001 | 0.28 | | (0.15, 0.42) | <0.001 |  |  |
| Education | 0.05 | (0.03, 0.07) | <0.001 | 0.05 | | (0.03, 0.07) | <0.001 |  |  |
| QPC-item 3 | 0.09 | (-0.04, 0.21) | 0.171 | 0.12 | | (0.00, 0.25) | 0.059 |  |  |
| Time | 0.02 | (-0.01, 0.05) | 0.284 | 0.03 | | (0.00, 0.05) | 0.028 |  |  |
| QPC-item 3 × time | -0.04 | (-0.08, -0.00) | 0.046 | -0.07 | | (-0.12, -0.03) | 0.001 |  |  |
| **Random Effects** | σ^2^ = 0.13, τ00 = 0.21, ICC = 0.61, Marginal R^2^ / Conditional R^2^ = 0.310 / 0.731 | | | σ^2^ = 0.13, τ00 = 0.21, ICC = 0.62, Marginal R^2^ / Conditional R^2^ = 0.311 / 0.739 | | | |  |  |
|  | 4. Forgetting about appointments | | | | | | | |  |
| (Intercept) | 1.81 | (1.05, 2.57) | <0.001 | 1.8 | | (1.01, 2.58) | <0.001 |  |  |
| Baseline age | -0.05 | (-0.05, -0.04) | <0.001 | -0.05 | | (-0.05, -0.04) | <0.001 |  |  |
| Sex | 0.31 | (0.17, 0.44) | <0.001 | 0.28 | | (0.14, 0.42) | <0.001 |  |  |
| Education | 0.05 | (0.03, 0.07) | <0.001 | 0.05 | | (0.03, 0.07) | <0.001 |  |  |
| QPC-item 4 | 0.08 | (-0.06, 0.22) | 0.258 | 0.07 | | (-0.10, 0.24) | 0.422 |  |  |
| Time | 0 | (-0.02, 0.02) | 0.863 | 0.01 | | (-0.01, 0.03) | 0.447 |  |  |
| QPC-item 4 × time | -0.05 | (-0.09, 0.00) | 0.058 | -0.05 | | (-0.11, 0.01) | 0.091 |  |  |
| **Random Effects** | σ^2^ = 0.13, τ00 = 0.21, ICC = 0.61, Marginal R^2^ / Conditional R^2^ = 0.307 / 0.730 | | | σ^2^ = 0.13, τ00 = 0.21, ICC = 0.62, Marginal R^2^ / Conditional R^2^ = 0.308 / 0.737 | | | |  |  |
|  | 5. Losing things | | | | | | | |  |
| (Intercept) | 1.82 | (1.06, 2.58) | <0.001 | 1.76 | | (0.97, 2.55) | <0.001 |  |  |
| Baseline age | -0.05 | (-0.05, -0.04) | <0.001 | -0.05 | | (-0.05, -0.04) | <0.001 |  |  |
| Sex | 0.31 | (0.17, 0.44) | <0.001 | 0.29 | | (0.15, 0.43) | <0.001 |  |  |
| Education | 0.05 | (0.03, 0.07) | <0.001 | 0.05 | | (0.03, 0.07) | <0.001 |  |  |
| QPC-item 5 | 0.12 | (-0.01, 0.26) | 0.079 | 0.07 | | (-0.09, 0.23) | 0.397 |  |  |
| Time | 0 | (-0.02, 0.02) | 0.977 | 0.01 | | (-0.01, 0.03) | 0.46 |  |  |
| QPC-item 5 × time | -0.03 | (-0.07, 0.02) | 0.208 | -0.04 | | (-0.09, 0.01) | 0.123 |  |  |
| **Random Effects** | σ^2^ = 0.13, τ00 = 0.21, ICC = 0.61, Marginal R^2^ / Conditional R^2^ = 0.309 / 0.732 | | | σ^2^ = 0.13, τ00 = 0.21, ICC = 0.62, Marginal R^2^ / Conditional R^2^ = 0.307 / 0.735 | | | |  |  |
|  | 6. Spatial orientation difficulties | | | | | | | |  |
| (Intercept) | 1.81 | (1.05, 2.57) | <0.001 | 1.77 | | (0.99, 2.54) | <0.001 |  |  |
| Baseline age | -0.05 | (-0.05, -0.04) | <0.001 | -0.04 | | (-0.05, -0.04) | <0.001 |  |  |
| Sex | 0.31 | (0.17, 0.44) | <0.001 | 0.3 | | (0.16, 0.44) | <0.001 |  |  |
| Education | 0.05 | (0.03, 0.07) | <0.001 | 0.05 | | (0.03, 0.07) | <0.001 |  |  |
| QPC-item 6 | 0.04 | (-0.12, 0.20) | 0.62 | 0 | | (-0.22, 0.23) | 0.991 |  |  |
| Time | 0 | (-0.02, 0.02) | 0.94 | 0.01 | | (-0.01, 0.03) | 0.317 |  |  |
| QPC-item 6 × time | -0.04 | (-0.10, 0.01) | 0.109 | -0.11 | | (-0.18, -0.03) | 0.005 |  |  |
| **Random Effects** | σ^2^ = 0.13, τ00 = 0.21, ICC = 0.61, Marginal R^2^ / Conditional R^2^ = 0.310 / 0.733 | | | σ^2^ = 0.12, τ00 = 0.20, ICC = 0.62, Marginal R^2^ / Conditional R^2^ = 0.319/ 0.739 | | | |  |  |
|  | 7. Forgetting about past experiences | | | | | | | |  |
| (Intercept) | 1.79 | (1.03, 2.55) | <0.001 | 1.78 | | (0.99, 2.57) | <0.001 |  |  |
| Baseline age | -0.05 | (-0.05, -0.04) | <0.001 | -0.05 | | (-0.05, -0.04) | <0.001 |  |  |
| Sex | 0.31 | (0.17, 0.44) | <0.001 | 0.28 | | (0.14, 0.42) | <0.001 |  |  |
| Education | 0.05 | (0.03, 0.07) | <0.001 | 0.05 | | (0.03, 0.07) | <0.001 |  |  |
| QPC-item 7 | 0 | (-0.14, 0.14) | 1 | 0.13 | | (-0.05, 0.31) | 0.17 |  |  |
| Time | 0 | (-0.02, 0.02) | 0.847 | 0.01 | | (-0.01, 0.03) | 0.329 |  |  |
| QPC-item 7 × time | -0.03 | (-0.08, 0.02) | 0.175 | -0.07 | | (-0.13, -0.02) | 0.014 |  |  |
| **Random Effects** | σ^2^ = 0.13, τ00 = 0.21, ICC = 0.61, Marginal R^2^ / Conditional R^2^ = 0.309 / 0.732 | | | σ^2^ = 0.13, τ00 = 0.21, ICC = 0.62, Marginal R^2^ / Conditional R^2^ = 0.308 / 0.740 | | | |  |  |
|  | 8. Word finding difficulties | | | | | | | |  |
| (Intercept) | 1.83 | (1.07, 2.59) | <0.001 | 1.73 | | (0.95, 2.52) | <0.001 |  |  |
| Baseline age | -0.05 | (-0.05, -0.04) | <0.001 | -0.05 | | (-0.05, -0.04) | <0.001 |  |  |
| Sex | 0.3 | (0.17, 0.44) | <0.001 | 0.29 | | (0.16, 0.43) | <0.001 |  |  |
| Education | 0.05 | (0.03, 0.07) | <0.001 | 0.05 | | (0.03, 0.07) | <0.001 |  |  |
| QPC-item 8 | -0.02 | (-0.15, 0.11) | 0.749 | 0.07 | | (-0.05, 0.20) | 0.249 |  |  |
| Time | 0 | (-0.04, 0.03) | 0.772 | 0.02 | | (-0.01, 0.04) | 0.193 |  |  |
| QPC-item 8 × time | 0 | (-0.05, 0.04) | 0.82 | -0.04 | | (-0.08, 0.00) | 0.061 |  |  |
| **Random Effects** | σ^2^ = 0.13, τ00 = 0.21, ICC = 0.61, Marginal R^2^ / Conditional R^2^ = 0.305 / 0.731 | | | σ^2^ = 0.13, τ00 = 0.21, ICC = 0.62, Marginal R^2^ / Conditional R^2^ = 0.311 / 0.736 | | | |  |  |
|  | 9. Limitation in daily activities | | | | | | | |  |
| (Intercept) | 1.85 | (1.09, 2.60) | <0.001 | 1.81 | | (1.04, 2.58) | <0.001 |  |  |
| Baseline age | -0.05 | (-0.05, -0.04) | <0.001 | -0.05 | | (-0.05, -0.04) | <0.001 |  |  |
| Sex | 0.3 | (0.16, 0.43) | <0.001 | 0.29 | | (0.15, 0.42) | <0.001 |  |  |
| Education | 0.05 | (0.03, 0.07) | <0.001 | 0.05 | | (0.03, 0.07) | <0.001 |  |  |
| QPC-item 9 | 0.09 | (-0.07, 0.25) | 0.292 | -0.08 | | (-0.26, 0.09) | 0.347 |  |  |
| Time | 0 | (-0.02, 0.02) | 0.829 | 0.02 | | (-0.00, 0.04) | 0.112 |  |  |
| QPC-item 9 × time | -0.08 | (-0.13, -0.03) | 0.003 | -0.08 | | (-0.14, -0.03) | 0.003 |  |  |
| **Random Effects** | σ^2^ = 0.13, τ00 = 0.20, ICC = 0.61, Marginal R^2^ / Conditional R^2^ = 0.316 / 0.733 | | | σ^2^ = 0.12, τ00 = 0.19, ICC = 0.61, Marginal R^2^ / Conditional R^2^ = 0.338 / 0.744 | | | |  |  |
|  | 10. Personality change | | | | | | | |  |
| (Intercept) | 1.77 | (1.00, 2.54) | <0.001 | 1.88 | | (1.10, 2.67) | <0.001 |  |  |
| Baseline age | -0.05 | (-0.05, -0.04) | <0.001 | -0.05 | | (-0.05, -0.04) | <0.001 |  |  |
| Sex | 0.31 | (0.17, 0.44) | <0.001 | 0.26 | | (0.12, 0.40) | <0.001 |  |  |
| Education | 0.05 | (0.03, 0.07) | <0.001 | 0.05 | | (0.03, 0.07) | <0.001 |  |  |
| QPC-item 10 | 0.16 | (0.04, 0.29) | 0.012 | -0.04 | | (-0.19, 0.12) | 0.646 |  |  |
| Time | 0.01 | (-0.01, 0.04) | 0.264 | 0.01 | | (-0.01, 0.03) | 0.384 |  |  |
| QPC-item 10 × time variable | -0.07 | (-0.11, -0.02) | 0.002 | -0.04 | | (-0.09, 0.01) | 0.081 |  |  |
| **Random Effects** | σ^2^ = 0.13, τ00 = 0.21, ICC = 0.63, Marginal R^2^ / Conditional R^2^ = 0.309 / 0.743 | | | σ^2^ = 0.13, τ00 = 0.20, ICC = 0.62, Marginal R^2^ / Conditional R^2^ = 0.314 / 0.736 | | | |  |  |
| **Note.** All models include fixed effects for age (baseline), sex, and education, as well as time, QPC-item, and the QPC-item × time interaction, and a random intercept for participant. Random effects are summarized per model as participant-level random intercept variance (τ00), residual variance (σ²), and intraclass correlation coefficient (ICC). Marginal and conditional R² are reported for completeness. Models were estimated using all available observations for each participant in order to maximize the use of available longitudinal data. | | | | | | | | | |

| **Supplementary Table 1c**  *Full linear mixed-effects model coefficients for QPC-item level models predicting memory composite score.* | | | | | | | | | | | |  |
| --- | --- | --- | --- | --- | --- | --- | --- | --- | --- | --- | --- | --- |
|  | **Patient** | | | | | **Informant** | | | | | |  |
| *Predictors* | *Estimates* | *95% CI* | *p* | *Estimates* | | | *95% CI* | | *p* | | |  |
|  | 1. Impression of memory change | | | | | | | | | | |  |
| (Intercept) | 0.91 | (-0.22, 2.05) | 0.115 | 0.71 | | | (-0.48, 1.91) | 0.242 | |  |  |  |
| Baseline age | -0.04 | (-0.05, -0.03) | <0.001 | -0.04 | | | (-0.05, -0.03) | <0.001 | |  |  |  |
| Sex | 0.47 | (0.26, 0.67) | <0.001 | 0.47 | | | (0.26, 0.68) | <0.001 | |  |  |  |
| Education | 0.05 | (0.02, 0.08) | 0.003 | 0.06 | | | (0.02, 0.09) | 0.002 | |  |  |  |
| QPC-item 1 | 0.05 | (-0.12, 0.22) | 0.589 | 0.41 | | | (0.21, 0.61) | <0.001 | |  |  |  |
| Time | 0.1 | (0.06, 0.14) | <0.001 | 0.13 | | | (0.10, 0.17) | <0.001 | |  |  |  |
| QPC-item 1 × time | -0.07 | (-0.13, -0.01) | 0.014 | -0.17 | | | (-0.23, -0.10) | <0.001 | |  |  |  |
| **Random Effects** | σ^2^ = 0.26, τ00 = 0.47, ICC = 0.64, Marginal R^2^ / Conditional R^2^ = 0.176 / 0.705 | | | | σ^2^ = 0.26, τ00 = 0.50, ICC = 0.66, Marginal R^2^ / Conditional R^2^ = 0.183 / 0.722 | | | | | |  |  |
|  | 2. Impression of worse memory in comparison to peers | | | | | | | | | | |  |
| (Intercept) | 1.02 | (-0.12, 2.16) | 0.079 | 0.97 | | | (-0.21, 2.15) | 0.108 | |  |  |  |
| Baseline age | -0.04 | (-0.05, -0.03) | <0.001 | -0.04 | | | (-0.05, -0.03) | <0.001 | |  |  |  |
| Sex | 0.47 | (0.26, 0.67) | <0.001 | 0.43 | | | (0.22, 0.64) | <0.001 | |  |  |  |
| Education | 0.05 | (0.02, 0.08) | 0.003 | 0.05 | | | (0.02, 0.09) | 0.002 | |  |  |  |
| QPC-item 2 | 0.01 | (-0.17, 0.19) | 0.946 | 0.19 | | | (-0.03, 0.42) | 0.088 | |  |  |  |
| Time | 0.1 | (0.06, 0.14) | <0.001 | 0.1 | | | (0.07, 0.14) | <0.001 | |  |  |  |
| QPC-item 2 × time | -0.06 | (-0.12, -0.00) | 0.039 | -0.15 | | | (-0.23, -0.07) | <0.001 | |  |  |  |
| **Random Effects** | σ^2^ = 0.27, τ00 = 0.46, ICC = 0.63, Marginal R^2^ / Conditional R^2^ = 0.180 / 0.696 | | | | σ^2^ = 0.26, τ00 = 0.48, ICC = 0.65, Marginal R^2^ / Conditional R^2^ = 0.182 / 0.711 | | | | | |  |  |
|  | 3. Difficulties with recalling past events | | | | | | | | | | |  |
| (Intercept) | 0.96 | (-0.18, 2.11) | 0.098 | 0.84 | | | (-0.35, 2.04) | 0.167 | |  |  |  |
| Baseline age | -0.04 | (-0.05, -0.03) | <0.001 | -0.04 | | | (-0.05, -0.03) | <0.001 | |  |  |  |
| Sex | 0.47 | (0.27, 0.68) | <0.001 | 0.45 | | | (0.24, 0.66) | <0.001 | |  |  |  |
| Education | 0.05 | (0.02, 0.08) | 0,003 | 0.05 | | | (0.02, 0.09) | 0.002 | |  |  |  |
| QPC-item 3 | 0.07 | (-0.11, 0.25) | 0.471 | 0.12 | | | (-0.07, 0.31) | 0.206 | |  |  |  |
| Time | 0.1 | (0.06, 0.14) | <0.001 | 0.1 | | | (0.07, 0.14) | <0.001 | |  |  |  |
| QPC-item 3 × time | -0.05 | (-0.11, 0.00) | 0.072 | -0.06 | | | (-0.12, 0.01) | 0.082 | |  | | |
| **Random Effects** | σ^2^ = 0.27, τ00 = 0.47, ICC = 0.64, Marginal R^2^ / Conditional R^2^ = 0.175 / 0.699 | | | | σ^2^ = 0.26, τ00 = 0.49, ICC = 0.65, Marginal R^2^ / Conditional R^2^ = 0.171 / 0.709 | | | | | |  |  |
|  | 4. Forgetting about appointments | | | | | | | | | | |  |
| (Intercept) | 0.92 | (-0.21, 2.06) | 0.111 | 0.94 | | | (-0.26, 2.13) | 0.124 | |  |  |  |
| Baseline age | -0.04 | (-0.05, -0.03) | <0.001 | -0.04 | | | (-0.05, -0.03) | <0.001 | |  |  |  |
| Sex | 0.48 | (0.28, 0.68) | <0.001 | 0.44 | | | (0.23, 0.65) | <0.001 | |  |  |  |
| Education | 0.05 | (0.02, 0.08) | 0.002 | 0.06 | | | (0.02, 0.09) | 0.002 | |  |  |  |
| QPC-item 4 | 0.17 | (-0.03, 0.37) | 0.096 | 0.15 | | | (-0.09, 0.39) | 0.216 | |  |  |  |
| Time | 0.08 | (0.05, 0.12) | <0.001 | 0.09 | | | (0.06, 0.12) | <0.001 | |  |  |  |
| QPC-item 4 × time | -0.06 | (-0.12, 0.01) | 0.101 | -0.08 | | | (-0.16, 0.01) | 0.078 | |  |  |  |
| **Random Effect** | σ^2^ = 0.27, τ00 = 0.47, ICC = 0.63, Marginal R^2^ / Conditional R^2^ = 0.175 / 0.698 | | | | σ^2^ = 0.26, τ00 = 0.49, ICC = 0.65, Marginal R^2^ / Conditional R^2^ = 0.174 / 0.715 | | | | | |  |  |
|  | 5. Losing things | | | | | | | | | | |  |
| (Intercept) | 0.98 | (-0.15, 2.11) | 0.09 | 0.87 | | | (-0.31, 2.06) | 0.149 | |  |  |  |
| Baseline age | -0.04 | (-0.05, -0.03) | <0.001 | -0.04 | | | (-0.05, -0.03) | <0.001 | |  |  |  |
| Sex | 0.48 | (0.27, 0.68) | <0.001 | 0.45 | | | (0.24, 0.66) | <0.001 | |  |  |  |
| Education | 0.05 | (0.02, 0.08) | 0.002 | 0.06 | | | (0.02, 0.09) | 0.002 | |  |  |  |
| QPC-item 5 | 0.3 | (0.10, 0.50) | 0.003 | 0.24 | | | (0.00, 0.47) | 0.049 | |  |  |  |
| Time | 0.09 | (0.06, 0.12) | <0.001 | 0.1 | | | (0.07, 0.13) | <0.001 | |  |  |  |
| QPC-item 5 × time | -0.07 | (-0.14, -0.01) | 0.03 | -0.1 | | | (-0.18, -0.03) | 0.006 | |  |  |  |
| **Random Effects** | σ^2^ = 0.27, τ00 = 0.47, ICC = 0.64, Marginal R^2^ / Conditional R^2^ = 0.180 / 0.701 | | | | σ^2^ = 0.26, τ00 = 0.49, ICC = 0.65, Marginal R^2^ / Conditional R^2^ = 0.175 / 0.709 | | | | | |  |  |
|  | 6. Spatial orientation difficulties | | | | | | | | | | |  |
| (Intercept) | 0.97 | (-0.17, 2.11) | 0.094 | 0.94 | | | (-0.24, 2.12) | 0.117 | |  |  |  |
| Baseline age | -0.04 | (-0.05, -0.03) | <0.001 | -0.04 | | | (-0.05, -0.03) | <0.001 | |  |  |  |
| Sex | 0.47 | (0.26, 0.67) | <0.001 | 0.46 | | | (0.25, 0.67) | <0.001 | |  |  |  |
| Education | 0.05 | (0.02, 0.08) | 0.002 | 0.05 | | | (0.02, 0.08) | 0.003 | |  |  |  |
| QPC-item 6 | 0.15 | (-0.08, 0.38) | 0.206 | -0.17 | | | (-0.50, 0.16) | 0.298 | |  |  |  |
| Time | 0.08 | (0.05, 0.11) | <0.001 | 0.09 | | | (0.06, 0.12) | <0.001 | |  |  |  |
| QPC-item 6 × time | -0.06 | (-0.13, 0.02) | 0.134 | -0.06 | | | (-0.16, 0.05) | 0.307 | |  |  |  |
| **Random Effects** | σ^2^ = 0.27, τ00 = 0.47, ICC = 0.64, Marginal R^2^ / Conditional R^2^ = 0.173 / 0.701 | | | | σ^2^ = 0.26, τ00 = 0.48, ICC = 0.65, Marginal R^2^ / Conditional R^2^ = 0.177 / 0.708 | | | | | |  |  |
|  | 7. Forgetting about past experiences | | | | | | | | | | |  |
| (Intercept) | 0.94 | (-0.20, 2.07) | 0.105 | 0.93 | | | (-0.25, 2.11) | 0.122 | |  |  |  |
| Baseline age | -0.04 | (-0.05, -0.03) | <0.001 | -0.04 | | | (-0.05, -0.03) | <0.001 | |  |  |  |
| Sex | 0.47 | (0.27, 0.68) | <0.001 | 0.44 | | | (0.23, 0.65) | <0.001 | |  |  |  |
| Education | 0.05 | (0.02, 0.09) | 0.002 | 0.05 | | | (0.02, 0.09) | 0.002 | |  |  |  |
| QPC-item 7 | -0.02 | (-0.22, 0.19) | 0.877 | 0.17 | | | (-0.09, 0.44) | 0.191 | |  |  |  |
| Time | 0.08 | (0.05, 0.11) | <0.001 | 0.1 | | | (0.07, 0.13) | <0.001 | |  |  |  |
| QPC-item 7 × time | -0.04 | (-0.11, 0.03) | 0.31 | -0.13 | | | (-0.21, -0.04) | 0.003 | |  |  |  |
| **Random Effects** | σ^2^ = 0.27, τ00 = 0.47, ICC = 0.63, Marginal R^2^ / Conditional R^2^ = 0.174 / 0.698 | | | | σ^2^ = 0.26, τ00 = 0.48, ICC = 0.65, Marginal R^2^ / Conditional R^2^ = 0.177 / 0.708 | | | | | |  |  |
|  | 8. Word finding difficulties | | | | | | | | | | |  |
| (Intercept) | 1.04 | (-0.10, 2.18) | 0.074 | 0.85 | | | (-0.34, 2.04) | 0.159 | |  |  |  |
| Baseline age | -0.04 | (-0.05, -0.03) | <0.001 | -0.04 | | | (-0.05, -0.03) | <0.001 | |  |  |  |
| Sex | 0.47 | (0.27, 0.68) | <0.001 | 0.47 | | | (0.26, 0.68) | <0.001 | |  |  |  |
| Education | 0.05 | (0.02, 0.08) | 0.002 | 0.06 | | | (0.02, 0.09) | 0.001 | |  |  |  |
| QPC-item 8 | -0.07 | (-0.26, 0.11) | 0.446 | 0.11 | | | (-0.07, 0.30) | 0.238 | |  |  |  |
| Time | 0.06 | (0.02, 0.11) | 0.008 | 0.1 | | | (0.06, 0.13) | <0.001 | |  |  |  |
| QPC-item 8 × time | 0.01 | (-0.05, 0.07) | 0.711 | -0.03 | | | (-0.09, 0.02) | 0.263 | |  |  |  |
| **Random Effects** | σ^2^ = 0.27, τ00 = 0.47, ICC = 0.64, Marginal R^2^ / Conditional R^2^ = 0.172 / 0.699 | | | | σ^2^ = 0.27, τ00 = 0.48, ICC = 0.64, Marginal R^2^ / Conditional R^2^ = 0.177 / 0.707 | | | | | |  |  |
|  | 9. Limitation in daily activities | | | | | | | | | | |  |
| (Intercept) | 1.04 | (-0.10, 2.17) | 0.074 | 0.84 | | | (-0.34, 2.03) | 0.164 | |  |  |  |
| Baseline age | -0.04 | (-0.05, -0.03) | <0.001 | -0.04 | | | (-0.05, -0.02) | <0.001 | |  |  |  |
| Sex | 0.46 | (0.26, 0.66) | <0.001 | 0.46 | | | (0.25, 0.66) | <0.001 | |  |  |  |
| Education | 0.05 | (0.02, 0.08) | 0.004 | 0.05 | | | (0.02, 0.09) | 0.002 | |  |  |  |
| QPC-item 9 | 0.05 | (-0.18, 0.28) | 0.692 | -0.02 | | | (-0.27, 0.24) | 0.899 | |  |  |  |
| Time | 0.08 | (0.05, 0.11) | <0.001 | 0.1 | | | (0.07, 0.13) | <0.001 | |  |  |  |
| QPC-item 9 × time | -0.07 | (-0.15, 0.00) | 0.064 | -0.11 | | | (-0.19, -0.03) | 0.006 | |  |  |  |
| **Random Effects** | σ^2^ = 0.27, τ00 = 0.47, ICC = 0.64, Marginal R^2^ / Conditional R^2^ = 0.177 / 0.703 | | | | σ^2^ = 0.26, τ00 = 0.47, ICC = 0.65, Marginal R^2^ / Conditional R^2^ = 0.188 / 0.712 | | | | | |  |  |
|  | 10. Personality change | | | | | | | | | | |  |
| (Intercept) | 0.9 | (-0.24, 2.05) | 0.122 | 1.03 | | | (-0.17, 2.22) | 0.092 | |  |  |  |
| Baseline age | -0.04 | (-0.05, -0.03) | <0.001 | -0.04 | | | (-0.05, -0.03) | <0.001 | |  |  |  |
| Sex | 0.48 | (0.27, 0.68) | <0.001 | 0.42 | | | (0.21, 0.63) | <0.001 | |  |  |  |
| Education | 0.05 | (0.02, 0.09) | 0.002 | 0.05 | | | (0.02, 0.09) | 0.003 | |  |  |  |
| QPC-item 10 | 0.19 | (0.00, 0.37) | 0.049 | -0.08 | | | (-0.31, 0.15) | 0.489 | |  |  |  |
| Time | 0.09 | (0.06, 0.12) | <0.001 | 0.09 | | | (0.05, 0.12) | <0.001 | |  |  |  |
| QPC-item 10 × time variable | -0.06 | (-0.12, 0.00) | 0.052 | -0.02 | | | (-0.10, 0.05) | 0.548 | |  |  |  |
| **Random Effects** | σ^2^ = 0.26, τ00 = 0.48, ICC = 0.64, Marginal R^2^ / Conditional R^2^ = 0.174 / 0.707 | | | | σ^2^ = 0.27, τ00 = 0.48, ICC = 0.64, Marginal R^2^ / Conditional R^2^ = 0.172 / 0.706 | | | | | |  |  |
| **Note.** All models include fixed effects for age (baseline), sex, and education, as well as time, QPC-item, and the QPC-item × time interaction, and a random intercept for participant. Random effects are summarized per model as participant-level random intercept variance (τ00), residual variance (σ²), and intraclass correlation coefficient (ICC). Marginal and conditional R² are reported for completeness. Models were estimated using all available observations for each participant in order to maximize the use of available longitudinal data. | | | | | | | | | | | |  |

| **Supplementary Table 1d**  *Full linear mixed-effects model coefficients for QPC-item level models predicting executive function/processing speed composite score.* | | | | | | | | | | | |  |  |
| --- | --- | --- | --- | --- | --- | --- | --- | --- | --- | --- | --- | --- | --- |
|  | **Patient** | | | | **Informant** | | | | | | | | |
| *Predictors* | *Estimates* | *95% CI* | *p* | | *Estimates* | *95% CI* | | *p* | |  |  |  |  |
|  | 1. Impression of memory change | | | | | | | | | | | | |
| (Intercept) | 2.65 | (1.81, 3.49) | <0.001 | | 2.57 | | (1.71, 3.42) | | <0.001 | |  |  |  |
| Baseline age | -0.05 | (-0.06, -0.04) | <0.001 | | -0.05 | | (-0.06, -0.04) | | <0.001 | |  |  |  |
| Sex | 0.22 | (0.07, 0.37) | 0.005 | | 0.21 | | (0.05, 0.36) | | 0.008 | |  |  |  |
| Education | 0.04 | (0.02, 0.07) | <0.001 | | 0.05 | | (0.02, 0.07) | | <0.001 | |  |  |  |
| QPC-item 1 | 0.05 | (-0.07, 0.17) | 0.376 | | 0.15 | | (0.01, 0.28) | | 0.034 | |  |  |  |
| Time | -0.05 | (-0.08, -0.02) | <0.001 | | -0.04 | | (-0.06, -0.02) | | 0.001 | |  |  |  |
| QPC-item 1 × time | -0.04 | (-0.08, -0.00) | 0.043 | | -0.09 | | (-0.13, -0.04) | | <0.001 | |  |  |  |
| **Random Effects** | σ^2^ = 0.13, τ00 = 0.26, ICC = 0.67, Marginal R^2^ / Conditional R^2^ = 0.333 / 0.781 | | | σ^2^ = 0.12, τ00 = 0.25, ICC = 0.68, Marginal R^2^ / Conditional R^2^ = 0.345 / 0.789 | | | | | | | | |  |
|  | 2. Impression of worse memory in comparison to peers | | | | | | | | | | | | |
| (Intercept) | 2.7 | (1.86, 3.54) | <0.001 | | 2.72 | | (1.87, 3.56) | | <0.001 | |  |  |  |
| Baseline age | -0.05 | (-0.06, -0.05) | <0.001 | | -0.05 | | (-0.06, -0.04) | | <0.001 | |  |  |  |
| Sex | 0.21 | (0.06, 0.36) | 0.006 | | 0.19 | | (0.04, 0.34) | | 0.012 | |  |  |  |
| Education | 0.05 | (0.02, 0.07) | <0.001 | | 0.04 | | (0.02, 0.07) | | <0.001 | |  |  |  |
| QPC-item 2 | 0.02 | (-0.11, 0.14) | 0.808 | | 0.05 | | (-0.11, 0.20) | | 0.531 | |  |  |  |
| Time | -0.05 | (-0.08, -0.03) | <0.001 | | -0.06 | | (-0.08, -0.03) | | <0.001 | |  |  |  |
| QPC-item 2 × time | -0.04 | (-0.07, 0.00) | 0.075 | | -0.08 | | (-0.13, -0.03) | | 0.003 | |  |  |  |
| **Random Effects** | σ^2^ = 0.13, τ00 = 0.26, ICC = 0.67, Marginal R^2^ / Conditional R^2^ = 0.339 / 0.779 | | | σ^2^ = 0.12, τ00 = 0.25, ICC = 0.67, Marginal R^2^ / Conditional R^2^ = 0.343 / 0.781 | | | | | | | | |  |
|  | 3. Difficulties with recalling past events | | | | | | | | | | | | |
| (Intercept) | 2.66 | (1.81, 3.50) | <0.001 | | 2.67 | | (1.81, 3.52) | | <0.001 | |  |  |  |
| Baseline age | -0.05 | (-0.06, -0.05) | <0.001 | | -0.05 | | (-0.06, -0.04) | | <0.001 | |  |  |  |
| Sex | 0.21 | (0.06, 0.37) | 0.005 | | 0.2 | | (0.05, 0.35) | | 0.011 | |  |  |  |
| Education | 0.05 | (0.02, 0.07) | <0.001 | | 0.04 | | (0.02, 0.07) | | <0.001 | |  |  |  |
| QPC-item 3 | 0.05 | (-0.08, 0.17) | 0.482 | | 0.04 | | (-0.08, 0.17) | | 0.507 | |  |  |  |
| Time | -0.06 | (-0.09, -0.03) | <0.001 | | -0.05 | | (-0.08, -0.02) | | <0.001 | |  |  |  |
| QPC-item 3 × time | -0.02 | (-0.06, 0.02) | 0.34 | | -0.05 | | (-0.10, -0.01) | | 0.014 | |  |  |  |
| **Random Effects** | σ^2^ = 0.13, τ00 = 0.26, ICC = 0.67, Marginal R^2^ / Conditional R^2^ = 0.332 / 0.780 | | | σ^2^ = 0.12, τ00 = 0.25, ICC = 0.67, Marginal R^2^ / Conditional R^2^ = 0.338 / 0.783 | | | | | | | | |  |
|  | 4. Forgetting about appointments | | | | | | | | | | | | |
| (Intercept) | 2.7 | (1.86, 3.54) | <0.001 | | 2.68 | | (1.83, 3.54) | | <0.001 | |  |  |  |
| Baseline age | -0.05 | (-0.06, -0.05) | <0.001 | | -0.05 | | (-0.06, -0.04) | | <0.001 | |  |  |  |
| Sex | 0.21 | (0.06, 0.36) | 0.006 | | 0.2 | | (0.05, 0.35) | | 0.008 | |  |  |  |
| Education | 0.05 | (0.02, 0.07) | <0.001 | | 0.05 | | (0.02, 0.07) | | <0.001 | |  |  |  |
| QPC-item 4 | -0.01 | (-0.15, 0.13) | 0.868 | | -0.05 | | (-0.22, 0.11) | | 0.525 | |  |  |  |
| Time | -0.07 | (-0.09, -0.05) | <0.001 | | -0.07 | | (-0.09, -0.05) | | <0.001 | |  |  |  |
| QPC-item 4 × time | -0.02 | (-0.07, 0.02) | 0.335 | | -0.01 | | (-0.07, 0.06) | | 0.855 | |  |  |  |
| **Random Effects** | σ^2^ = 0.13, τ00 = 0.26, ICC = 0.67, Marginal R^2^ / Conditional R^2^ = 0.330 / 0.780 | | | σ^2^ = 0.13, τ00 = 0.26, ICC = 0.67, Marginal R^2^ / Conditional R^2^ = 0.333 / 0.779 | | | | | | | | |  |
|  | 5. Losing things | | | | | | | | | | | | |
| (Intercept) | 2.73 | (1.89, 3.56) | <0.001 | | 2.66 | | (1.80, 3.52) | | <0.001 | |  |  |  |
| Baseline age | -0.05 | (-0.06, -0.04) | <0.001 | | -0.05 | | (-0.06, -0.04) | | <0.001 | |  |  |  |
| Sex | 0.2 | (0.05, 0.35) | 0.008 | | 0.21 | | (0.05, 0.36) | | 0.008 | |  |  |  |
| Education | 0.04 | (0.02, 0.07) | <0.001 | | 0.04 | | (0.02, 0.07) | | <0.001 | |  |  |  |
| QPC-item 5 | -0.08 | (-0.22, 0.06) | 0.252 | | -0.01 | | (-0.18, 0.15) | | 0.863 | |  |  |  |
| Time | -0.08 | (-0.10, -0.05) | <0.001 | | -0.07 | | (-0.09, -0.05) | | <0.001 | |  |  |  |
| QPC-item 5 × time | 0.01 | (-0.03, 0.06) | 0.533 | | 0.01 | | (-0.04, 0.06) | | 0.726 | |  |  |  |
| **Random Effects** | σ^2^ = 0.13, τ00 = 0.26, ICC = 0.67, Marginal R^2^ / Conditional R^2^ = 0.330 / 0.779 | | | σ^2^ = 0.13, τ00 = 0.26, ICC = 0.67, Marginal R^2^ / Conditional R^2^ = 0.329 / 0.780 | | | | | | | | |  |
|  | 6. Spatial orientation difficulties | | | | | | | | | | | | |
| (Intercept) | 2.71 | (1.87, 3.54) | <0.001 | | 2.67 | | (1.82, 3.51) | | <0.001 | |  |  |  |
| Baseline age | -0.05 | (-0.06, -0.04) | <0.001 | | -0.05 | | (-0.06, -0.04) | | <0.001 | |  |  |  |
| Sex | 0.22 | (0.07, 0.37) | 0.005 | | 0.22 | | (0.07, 0.37) | | 0.004 | |  |  |  |
| Education | 0.04 | (0.02, 0.07) | <0.001 | | 0.04 | | (0.02, 0.07) | | 0.001 | |  |  |  |
| QPC-item 6 | -0.09 | (-0.26, 0.07) | 0.265 | | -0.1 | | (-0.33, 0.13) | | 0.396 | |  |  |  |
| Time | -0.07 | (-0.09, -0.05) | <0.001 | | -0.06 | | (-0.08, -0.04) | | <0.001 | |  |  |  |
| QPC-item 6 × time | -0.01 | (-0.06, 0.05) | 0.784 | | -0.07 | | (-0.15, 0.00) | | 0.054 | |  |  |  |
| **Random Effects** | σ^2^ = 0.13, τ00 = 0.26, ICC = 0.67, Marginal R^2^ / Conditional R^2^ = 0.334 / 0.780 | | | σ^2^ = 0.12, τ00 = 0.25, ICC = 0.67, Marginal R^2^ / Conditional R^2^ = 0.344 / 0.786 | | | | | | | | |  |
|  | 7. Forgetting about past experiences | | | | | | | | | | | | |
| (Intercept) | 2.69 | (1.85, 3.53) | <0.001 | | 2.68 | | (1.82, 3.54) | | <0.001 | |  |  |  |
| Baseline age | -0.05 | (-0.06, -0.04) | <0.001 | | -0.05 | | (-0.06, -0.04) | | <0.001 | |  |  |  |
| Sex | 0.21 | (0.06, 0.36) | 0.006 | | 0.21 | | (0.05, 0.36) | | 0.008 | |  |  |  |
| Education | 0.04 | (0.02, 0.07) | <0.001 | | 0.04 | | (0.02, 0.07) | | <0.001 | |  |  |  |
| QPC-item 7 | -0.05 | (-0.19, 0.10) | 0.514 | | -0.03 | | (-0.21, 0.15) | | 0.734 | |  |  |  |
| Time | -0.07 | (-0.09, -0.05) | <0.001 | | -0.07 | | (-0.09, -0.04) | | <0.001 | |  |  |  |
| QPC-item 7 × time | -0.01 | (-0.06, 0.04) | 0.769 | | 0 | | (-0.06, 0.06) | | 0.922 | |  |  |  |
| **Random Effects** | σ^2^ = 0.13, τ00 = 0.26, ICC = 0.67, Marginal R^2^ / Conditional R^2^ = 0.331 / 0.779 | | | σ^2^ = 0.13, τ00 = 0.26, ICC = 0.67, Marginal R^2^ / Conditional R^2^ = 0.333 / 0.780 | | | | | | | | |  |
|  | 8. Word finding difficulties | | | | | | | | | | | | |
| (Intercept) | 2.71 | (1.87, 3.55) | <0.001 | | 2.64 | | (1.78, 3.49) | | <0.001 | |  |  |  |
| Baseline age | -0.05 | (-0.06, -0.05) | <0.001 | | -0.05 | | (-0.06, -0.04) | | <0.001 | |  |  |  |
| Sex | 0.21 | (0.06, 0.36) | 0.007 | | 0.21 | | (0.06, 0.36) | | 0.007 | |  |  |  |
| Education | 0.04 | (0.02, 0.07) | <0.001 | | 0.04 | | (0.02, 0.07) | | <0.001 | |  |  |  |
| QPC-item 8 | 0.03 | (-0.10, 0.16) | 0.642 | | 0.03 | | (-0.10, 0.16) | | 0.635 | |  |  |  |
| Time | -0.06 | (-0.09, -0.03) | <0.001 | | -0.05 | | (-0.08, -0.02) | | <0.001 | |  |  |  |
| QPC-item 8 × time | -0.02 | (-0.06, 0.02) | 0.385 | | -0.04 | | (-0.08, 0.00) | | 0.062 | |  |  |  |
| **Random Effects** | σ^2^ = 0.13, τ00 = 0.26, ICC = 0.67, Marginal R^2^ / Conditional R^2^ = 0.330 / 0.779 | | | σ^2^ = 0.13, τ00 = 0.25, ICC = 0.67, Marginal R^2^ / Conditional R^2^ = 0.340 / 0.781 | | | | | | | | |  |
|  | 9. Limitation in daily activities | | | | | | | | | | | | |
| (Intercept) | 2.71 | (1.87, 3.54) | <0.001 | | 2.72 | | (1.86, 3.58) | | <0.001 | |  |  |  |
| Baseline age | -0.05 | (-0.06, -0.04) | <0.001 | | -0.05 | | (-0.06, -0.04) | | <0.001 | |  |  |  |
| Sex | 0.2 | (0.05, 0.35) | 0.008 | | 0.21 | | (0.06, 0.36) | | 0.007 | |  |  |  |
| Education | 0.04 | (0.02, 0.07) | <0.001 | | 0.04 | | (0.02, 0.07) | | 0.001 | |  |  |  |
| QPC-item 9 | 0.05 | (-0.11, 0.21) | 0.548 | | -0.06 | | (-0.24, 0.12) | | 0.526 | |  |  |  |
| Time | -0.06 | (-0.09, -0.04) | <0.001 | | -0.06 | | (-0.08, -0.04) | | <0.001 | |  |  |  |
| QPC-item 9 × time | -0.05 | (-0.11, -0.00) | 0.048 | | -0.05 | | (-0.10, 0.01) | | 0.103 | |  |  |  |
| **Random Effects** | σ^2^ = 0.13, τ00 = 0.26, ICC = 0.67, Marginal R^2^ / Conditional R^2^ = 0.334 / 0.777 | | | σ^2^ = 0.12, τ00 = 0.25, ICC = 0.67, Marginal R^2^ / Conditional R^2^ = 0.344 / 0.783 | | | | | | | | |  |
|  | 10. Personality change | | | | | | | | | | | | |
| (Intercept) | 2.75 | (1.92, 3.59) | <0.001 | | 2.72 | | (1.86, 3.57) | | <0.001 | |  |  |  |
| Baseline age | -0.06 | (-0.06, -0.05) | <0.001 | | -0.05 | | (-0.06, -0.04) | | <0.001 | |  |  |  |
| Sex | 0.21 | (0.06, 0.36) | 0.006 | | 0.19 | | (0.04, 0.34) | | 0.014 | |  |  |  |
| Education | 0.04 | (0.02, 0.07) | <0.001 | | 0.04 | | (0.02, 0.07) | | <0.001 | |  |  |  |
| QPC-item 10 | 0.04 | (-0.09, 0.17) | 0.516 | | 0.01 | | (-0.14, 0.17) | | 0.859 | |  |  |  |
| Time | -0.06 | (-0.08, -0.04) | <0.001 | | -0.05 | | (-0.08, -0.03) | | <0.001 | |  |  |  |
| QPC-item 10 × time variable | -0.04 | (-0.09, -0.00) | 0.041 | | -0.06 | | (-0.11, -0.01) | | 0.019 | |  |  |  |
| **Random Effects** | σ^2^ = 0.13, τ00 = 0.26, ICC = 0.67, Marginal R^2^ / Conditional R^2^ = 0.335 / 0.783 | | | σ^2^ = 0.12, τ00 = 0.25, ICC = 0.67, Marginal R^2^ / Conditional R^2^ = 0.342 / 0.785 | | | | | | | | |  |
| **Note.** All models include fixed effects for age (baseline), sex, and education, as well as time, QPC-item, and the QPC-item × time interaction, and a random intercept for participant. Random effects are summarized per model as participant-level random intercept variance (τ00), residual variance (σ²), and intraclass correlation coefficient (ICC). Marginal and conditional R² are reported for completeness. Models were estimated using all available observations for each participant in order to maximize the use of available longitudinal data. | | | | | | | | | | | |  |  |

| **Supplementary Table 2.**  *Model fit statistics for linear mixed-effects models predicting composite cognitive outcomes.* | | | | | | | |
| --- | --- | --- | --- | --- | --- | --- | --- |
| **Outcome** | **Model type** | **Source of Report** | **AIC** | **BIC** | **R² (marg.)** | **R² (cond.)** | **ICC** |
| **Global cognition** | QPC-total score | Patient | 1182.9 | 1225.7 | 0.318 | 0.740 | 0.618 |
|  |  | Informant | 1056.0 | 1097.9 | 0.336 | 0.749 | 0.621 |
|  | QPC-item level (range) | Patient | 1165.3 – 1195.9 | 1207.9 – 1238.6 | 0.305 – 0.316 | 0.730 – 0.743 | 0.607 – 0.627 |
|  |  | Informant | 1044.9 – 1090.4 | 1086.8 – 1132.3 | 0.307 – 0.338 | 0.735 – 0.753 | 0.613 – 0.635 |
| **Memory** | QPC-total score | Patient | 1818.7 | 1861.4 | 0.181 | 0.705 | 0.640 |
|  |  | Informant | 1665.5 | 1707.4 | 0.186 | 0.713 | 0.648 |
|  | QPC-item level (range) | Patient | 1792.5 – 1822.2 | 1835.0 – 1864.9 | 0.172 – 0.180 | 0.696 – 0.707 | 0.630 – 0.645 |
|  |  | Informant | 1645.5 – 1671.0 | 1687.4 – 1712.9 | 0.171 – 0.188 | 0.706 – 0.722 | 0.644 – 0.660 |
| **Executive function/**  **processing speed** | QPC-total score | Patient | 1230.5 | 1273.3 | 0.339 | 0.783 | 0.672 |
|  |  | Informant | 1103.6 | 1145.5 | 0.352 | 0.787 | 0.671 |
|  | QPC-item level (range) | Patient | 1214.6 – 1234.2 | 1257.2 – 1276.9 | 0.330 – 0.335 | 0.777 – 0.783 | 0.666 – 0.673 |
|  |  | Informant | 1095.2 – 1122.4 | 1137.1 – 1164.3 | 0.329 – 0.345 | 0.779 – 0.789 | 0.667 – 0.678 |
| **Note.** Fit statistics are reported for linear mixed-effects models with composite cognitive outcome scores including fixed effects for age, sex, and education, and a random intercept for participant. R² (marg.) and R² (cond.) denote marginal and conditional R², respectively; AIC, Akaike Information Criterion; BIC, Bayesian Information Criterion; ICC, intraclass correlation coefficient. For item-level analyses, fit statistics are reported as ranges across the ten individual QPC item models. | | | | | | | |

| **Supplementary Table S3a.**  *Sensitivity Analysis: Linear Mixed Effect Models Predicting Decline in Global Cognitive Composite Z-Score (restricted sample).* | | | | | | | | | | | | | |
| --- | --- | --- | --- | --- | --- | --- | --- | --- | --- | --- | --- | --- | --- |
|  |  | **PATIENT (n=237)** | | | | | | **INFORMANT (n=237)** | | | | | |
|  | **Global cognitive composite z-score** | Item  (95% CI) | p | Time variable  (95% CI) | p | **Item*time variable**  (95% CI) | **p** | Item  (95% CI) | p | Time variable  (95% CI) | p | **Item*time variable**  (95% CI) | **p** |
|  |  | Estimates (CI) | | | | | | Estimates (CI) | | | | | |
| 1 | Impression of memory change^##, §§^ | 0,07  (-0.05, 0.19) | 0.267 | 0.02  (-0.00, 0.05) | 0.101 | **-0.06**  **(-0.10, -0.02)** | **0.006** | 0.28  (0.15, 0.41) | <0.001 | 0.05 (0.02, 0.07) | <0.001 | **-0.14**  **(-0.18, -0.09)** | **<0.001*** |
| 2 | Impression of worse memory in comparison to peers^#, §§^ | 0,04  (-0.09, 0.16) | 0.576 | 0.02  (-0.01, 0.05) | 0.251 | **-0.04**  **(-0.08, -0.00)** | **0.032** | 0.16 (0.01, 0.31) | 0.038 | 0.02 (0.00, 0.05) | 0.03 | **-0.14**  **(-0.19, -0.09)** | **<0.001*** |
| 3 | Difficulties with recalling past events^###, §§§^ | 0.09  (-0.04, 0.22) | 0.169 | 0.02  (-0.01, 0.05) | 0.203 | **-0.04**  **(-0.08, 0.00)** | **0.055** | 0.13  (0.00, 0.26) | 0.046 | 0.03  (0.01, 0.06) | 0.018 | **-0.07**  **(-0.11, -0.03)** | **0.001*** |
| 4 | Forgetting about appointments^###, §§§§§^ | 0.09  (-0.06, 0.23) | 0.239 | 0.01  (-0.02, 0.03) | 0.642 | -0.04  (-0.09, 0.00) | 0.076 | 0.06  (-0.10, 0.23) | 0.469 | 0.01  (-0.01, 0.03) | 0.384 | -0.04  (-0.10, 0.02) | 0.242 |
| 5 | Losing things^####, §§§§^ | 0.14  (-0.01, 0.28) | 0.062 | 0.01  (-0.02, 0.03) | 0.633 | -0.03  (-0.08, 0.01) | 0.139 | 0.07  (-0.09, 0.23) | 0.408 | 0.01  (-0.01, 0.03) | 0.306 | -0.04  (-0.09, 0.01) | 0.099 |
| 6 | Spatial orientation difficulties^######, §§§§§§^ | 0.04  (-0.13, 0.21) | 0.628 | 0  (0) | 0.831 | -0.04  (-0.10, 0.01) | 0.152 | 0  (-0.22, 0.22) | 0.986 | 0.01  (-0.01, 0.03) | 0.201 | **-0.11**  **(-0.18, -0.03)** | **0.004*** |
| 7 | Forgetting about past experiences^#####, §§§§§^ | 0.03  (-0.12, 0.18) | 0.686 | 0  (-0.02, 0.02) | 0.943 | -0.03  (-0.08, 0.02) | 0.309 | 0.14  (-0.04, 0.32) | 0.134 | 0.01  (-0.01, 0.03) | 0.24 | **-0.07**  **(-0.13, -0.01)** | **0.017** |
| 8 | Word finding difficulties^#####, §^ | -0.01  (-0.14, 0.12) | 0.861 | 0  (-0.03, 0.03) | 0.974 | -0.01  (-0.05, 0.04) | 0.800 | 0.06  (-0.06, 0.19) | 0.325 | 0.02  (-0.01, 0.05) | 0.14 | -0.04  (-0.08, 0.00) | 0.067 |
| 9 | Limitation in daily activities^####, §§§^ | 0.08  (-0.09, 0.25) | 0.336 | 0.01  (-0.01, 0.03) | 0.567 | **-0.08**  **(-0.14, -0.03)** | **0.004*** | -0.1  (-0.28, 0.07) | 0.24 | 0.02  (-0.00, 0.04) | 0.064 | **-0.08**  **(-0.13, -0.03)** | **0.003*** |
| 10 | Personality change^###, §§^ | 0.19  (0.06, 0.32) | 0.004 | 0.02  (-0.00, 0.04) | 0.103 | **-0.07**  **(-0.12, -0.03)** | **0.001*** | -0.04  (-0.19, 0.12) | 0.629 | 0.01  (-0.01, 0.04) | 0.255 | -0.05  (-0.10, 0.00) | 0.071 |
| **Total score**^#######, §§§§§§§^ | | 0.03  (-0.00, 0.06) | 0.094 | 0.05  (0.02, 0.09) | **0.004** | **-0.02**  **(-0.03, -0.01)** | **<0.001*** | 0.02  (-0.01, 0.04) | 0.303 | 0.05  (0.02, 0.08) | <0.001 | **-0.02**  **(-0.03, -0.01)** | **<0.001*** |
| **Note.** Cells shaded in grey and shown in bold indicate statistically significant results exclusively for the item*time interaction; * denotes results that remained statistically significant after Bonferroni correction (p<0.005); analyses controlled for sex, age, and education; analyses were restricted to participants with both patient- and informant-reported baseline questionnaires available; within this restricted sample, linear mixed-effects models used all available repeated follow-up observations; number of observations in self-reported QPC version: ^#^ 782; ^##^ 789; ^###^ 790; ^####^ 793; ^#####^ 794; ^######^ 796; ^#######^ 799; number of observations in informant-reported QPC version: ^§^ 751; ^§§^ 752; ^§§§^ 753; ^§§§§^ 754; ^§§§§§^ 755; ^§§§§§§^ 757, ^§§§§§§§^ 759. | | | | | | | | | | | | | |

| **Supplementary Table S3b.**  *Sensitivity Analysis: Linear Mixed Effect Models Predicting Decline in Memory Composite Z-Score (restricted sample).* | | | | | | | | | | | | | |
| --- | --- | --- | --- | --- | --- | --- | --- | --- | --- | --- | --- | --- | --- |
|  |  | **PATIENT (n=237)** | | | | | | **INFORMANT (n=237)** | | | | | |
|  | **Memory composite**  **z-score** | Item  (95% CI) | p  (95% CI) | Time variable  (95% CI) | p | **Item*time variable**  (95% CI) | **p** | Item  (95% CI) | p | Time variable  (95% CI) | p | **Item*time variable**  (95% CI) | **p** |
|  |  | Estimates (CI) | | | | | | Estimates (CI) | | | | | |
| 1 | Impression of memory change^##, §§^ | 0.06  (-0.11, 0.24) | 0.486 | 0.11  (0.07, 0.15) | <0.001 | **-0.07**  **(-0.13, -0.02)** | **0.013** | 0.41 (0.21 – 0.60) | <0.001 | 0.14 (0.10, 0.17) | <0.001 | **-0.17**  **(-0.23, -0.10)** | **<0.001*** |
| 2 | Impression of worse memory in comparison to peers^#, §§^ | 0  (-0.19, 0.18) | 0.977 | 0.1  (0.06, 0.14) | <0.001 | **-0.05**  **(-0.11, 0.00)** | **0.067** | 0.19  (-0.04, 0.41) | 0.102 | 0.11 (0.08, 0.14) | <0.001 | **-0.15**  **(-0.23, -0.07)** | **<0.001*** |
| 3 | Difficulties with recalling past events^###, §§§^ | 0.06  (-0.13, 0.25) | 0.528 | 0.1 (0.06, 0.15) | <0.001 | -0.05  (-0.11, 0.00) | 0.091 | 0.14  (-0.05, 0.32) | 0.151 | 0.11 (0.07, 0.14) | <0.001 | -0.05  (-0.12, 0.01) | 0.094 |
| 4 | Forgetting about appointments^###, §§§§§^ | 0.2  (-0.01, 0.40) | 0.063 | 0.09 (0.06, 0.12) | <0.001 | -0.06  (-0.13, 0.01) | 0.091 | 0.13  (-0.11, 0.37) | 0.274 | 0.09 (0.06, 0.13) | <0.001 | -0.06  (-0.15, 0.03) | 0.169 |
| 5 | Losing things^####, §§§§^ | 0.33  (0.12, 0.54) | 0.002 | 0.1  (0.07, 0.13) | <0.001 | **-0.08**  **(-0.15, -0.02)** | **0.013** | 0.23  (-0.00, 0.47) | 0.051 | 0.1 (0.07, 0.14) | <0.001 | **-0.11**  **(-0.18, -0.03)** | **0.004*** |
| 6 | Spatial orientation difficulties^######, §§§§§§^ | 0.15  (-0.09, 0.39) | 0.227 | 0.09 (0.06, 0.12) | <0.001 | -0.05  (-0.13, 0.03) | 0.191 | -0.19  (-0.51, 0.14) | 0.264 | 0.09 (0.06, 0.12) | <0.001 | -0.06  (-0.16, 0.05) | 0.283 |
| 7 | Forgetting about past experiences^#####, §§§§^ | 0.01  (-0.20, 0.23) | 0.91 | 0.08  (0.05, 0.11) | <0.001 | -0.02  (-0.10, 0.05) | 0.504 | 0.2  (-0.06, 0.47) | 0.126 | 0.1 (0.07, 0.13) | <0.001 | **-0.13**  **(-0.21, -0.04)** | **0.003*** |
| 8 | Word finding difficulties^#####, §^ | -0.04  (-0.23, 0.15) | 0.68 | 0.07 (0.03, 0.12) | 0.002 | 0.01  (-0.05, 0.06) | 0.859 | 0.09  (-0.09, 0.28) | 0.32 | 0.1 (0.06, 0.14) | <0.001 | -0.03  (-0.09, 0.03) | 0.291 |
| 9 | Limitation in daily activities^####, §§§^ | 0.03  (-0.22, 0.27) | 0.84 | 0.09 (0.06, 0.12) | <0.001 | -0.07  (-0.15, 0.01) | 0.072 | -0.03  (-0.28, 0.23) | 0.846 | 0.1 (0.07, 0.14) | <0.001 | **-0.11**  **(-0.19, -0.03)** | **0.006** |
| 10 | Personality change^###, §§^ | 0.2 (0.01, 0.39) | 0.039 | 0.1 (0.06, 0.13) | <0.001 | **-0.06**  **(-0.13, -0.00)** | **0.039** | -0.08  (-0.31, 0.15) | 0.506 | 0.09 (0.06, 0.12) | <0.001 | -0.02  (-0.10, 0.05) | 0.524 |
| **Total score**^#######, §§§§§§§^ | | 0.04  (-0.01, 0.08) | 0.109 | 0.15  (0.10, 0.20) | <0.001 | **-0.02**  **(-0.03, -0.01)** | **0.001*** | 0.03  (-0.01, 0.08) | 0,159 | 0.14  (0.10, 0.18) | <0.001 | **-0.03**  **(-0.04, -0.01)** | **<0.001*** |
| **Note.** Cells shaded in grey and shown in bold indicate statistically significant results exclusively for the item*time interaction; * denotes results that remained statistically significant after Bonferroni correction (p<0.005); analyses controlled for sex, age, and education; analyses were restricted to participants with both patient- and informant-reported baseline questionnaires available; within this restricted sample, linear mixed-effects models used all available repeated follow-up observations; number of observations in self-reported QPC version: ^#^ 781; ^##^ 788; ^###^ 789; ^####^ 792; ^#####^ 793; ^######^ 795; ^#######^ 798; number of observations in informant-reported QPC version: ^§^ 750; ^§§^ 751; ^§§§^ 752; ^§§§§^ 753; ^§§§§§^ 754; ^§§§§§§^ 756; ^§§§§§§§^ 758. | | | | | | | | | | | | | |

| **Supplementary Table S3c.**  *Sensitivity Analysis: Linear Mixed Effect Models Predicting Decline in Executive Function/Processing Speed Composite Z-Score (restricted sample).* | | | | | | | | | | | | | |
| --- | --- | --- | --- | --- | --- | --- | --- | --- | --- | --- | --- | --- | --- |
|  |  |  | **PATIENT (n=237)** | | |  |  |  | **INFORMANT (n=237)** | | |  |  |
|  | **Executive – processing speed composite z-score** | Item  (95% CI) | p | Time variable  (95% CI) | p | **Item*time variable**  (95% CI) | p | Item  (95% CI) | p | Time variable  (95% CI) | p | **Item*time variable**  (95% CI) | p |
|  |  | Estimates (CI) | | | | | | Estimates (CI) | | | | | |
| 1 | Impression of memory change ^##, §§^ | 0.07  (-0.05, 0.20) | 0.262 | -0.05  (-0.08, -0.02) | <0.001 | **-0.04**  **(-0.08, -0.00)** | **0.042** | 0.15 (0.01, 0.29) | 0.034 | -0.04  (-0.06, -0.02) | 0.001 | **-0.09**  **(-0.13, -0.05)** | **<0.001*** |
| 2 | Impression of worse memory in comparison to peers^#, §§^ | 0.03  (-0.10, 0.16) | 0.684 | -0.05  (-0.08, -0.02) | <0.001 | -0.04  (-0.08, 0.00) | 0.070 | 0.04  (-0.12, 0.19) | 0.652 | -0.06  (-0.08, -0.03) | <0.001 | **-0.08**  **(-0.13, -0.03)** | **0.004*** |
| 3 | Difficulties with recalling past events^###, §§§^ | 0.05  (-0.08, 0.19) | 0.422 | -0.06  (-0.09, -0.03) | <0.001 | -0.02  (-0.06, 0.02) | 0.324 | 0.04  (-0.09, 0.17) | 0.528 | -0.05  (-0.07, -0.02) | <0.001 | **-0.05**  **(-0.10, -0.01)** | **0.017** |
| 4 | Forgetting about appointments^###, §§§§§^ | 0.04  (-0.18, 0.11) | 0.618 | -0.07  (-0.09, -0.05) | <0.001 | -0.02  (-0.07, 0.03) | 0.500 | -0.06  (-0.23, 0.11) | 0.463 | -0.07  (-0.09, -0.05) | <0.001 | 0.01  (-0.05, 0.07) | 0.817 |
| 5 | Losing things^####, §§§§^ | -0.07  (-0.22, 0.08) | 0.348 | -0.08  (-0.10, -0.05) | <0.001 | 0.01  (-0.03, 0.06) | 0.563 | -0.02  (-0.18, 0.15) | 0.843 | -0.07  (-0.09, -0.05) | <0.001 | 0.01  (-0.04, 0.06) | 0.749 |
| 6 | Spatial orientation difficulties^###### , §§§§§§^ | -0.09  (-0.27, 0.08) | 0.284 | -0.07  (-0.09, -0.05) | <0.001 | 0.00  (-0.06, 0.05) | 0.875 | -0.09  (-0.32, 0.13) | 0.415 | -0.06  (-0.08, -0.04) | <0.001 | -0.07  (-0.15, 0.00) | 0.055 |
| 7 | Forgetting about past experiences^#####, §§§§§^ | -0.03  (-0.19, 0.12) | 0.679 | -0.07  (-0.09, -0.05) | <0.001 | -0.01  (-0.06, 0.05) | 0.796 | -0.04  (-0.22, 0.15) | 0.703 | -0.07  (-0.09, -0.04) | <0.001 | 0.00  (-0.06, 0.06) | 0.902 |
| 8 | Word finding difficulties^#####, §^ | 0.04  (-0.10, 0.17) | 0.602 | -0.06  (-0.10, -0.03) | <0.001 | -0.01  (-0.06, 0.03) | 0.488 | 0.03  (-0.10, 0.15) | 0.694 | -0.05  (-0.08, 0.02) | <0.001 | -0.04  (-0.08, 0.01) | 0.087 |
| 9 | Limitation in daily activities^####, §§§^ | 0.07  (-0.10, 0.25) | 0.423 | -0.06  (-0.09, -0.04) | <0.001 | **-0.06**  **(-0.12, -0.01)** | **0.027** | -0.07  (-0.25, 0.11) | 0.442 | -0.06  (-0.08, -0.04) | <0.001 | -0.04  (-0.10, 0.01) | 0.119 |
| 10 | Personality change^###, §§^ | 0.08  (-0.05, 0.22) | 0.226 | -0.06  (-0.08, -0.03) | <0.001 | **-0.06**  **(-0.10, -0.01)** | **0.01** | 0.01  (-0.14, 0.17) | 0.87 | -0.05  (-0.08, -0.03) | <0.001 | **-0.06**  **(-0.11, -0.01)** | **0.018** |
| **Total score**^#######, §§§§§§§^ | | 0  (-0.04, 0.03) | 0.789 | -0.04  (-0.08, -0.01) | 0.021 | **-0.01**  **(-0.02, -0.00)** | **0.043** | -0.01  (-0.04, 0.02) | 0.501 | -0.04  (-0.07, -0.01) | 0.004 | **-0.01**  **(-0.02, -0.00)** | **0.006** |
| **Note.** Cells shaded in grey and shown in bold indicate statistically significant results exclusively for the item*time interaction; * denotes results that remained statistically significant after Bonferroni correction (p<0.005); analyses controlled for sex, age, and education; analyses were restricted to participants with both patient- and informant-reported baseline questionnaires available; within this restricted sample, linear mixed-effects models used all available repeated follow-up observations; number of observations in self-reported QPC version: ^#^ 782; ^##^ 789; ^###^ 790; ^####^ 793; ^#####^ 794; ^######^ 796; ^#######^ 799; number of observations in informant-reported QPC version: ^§^ 751; ^§§^ 752; ^§§§^ 753; ^§§§§^ 754; ^§§§§§^ 755; ^§§§§§§^ 757; ^§§§§§§§^ 759. | | | | | | | | | | | | | |
